# Harnessing Electrocatalytic Coupling of Carbon Dioxide and Methanol for High‐Efficiency Formic Acid Production

**DOI:** 10.1002/anie.202512078

**Published:** 2025-09-06

**Authors:** Zhikeng Zheng, Xiaobo Zheng, Ligang Wang, Huiming Wen, Ke Li, Zhenhao Xu, Yameng Fan, Peng Li, Suyu Zhang, Bin Liu, Dingsheng Wang, Kai Yan, Guoxiu Wang

**Affiliations:** ^1^ School of Environmental Science and Engineering Sun Yat‐sen University Guangzhou 510275 China; ^2^ Center for Clean Energy Technology School of Mathematical and Physical Sciences Faculty of Science University of Technology Sydney Sydney New South Wales 2007 Australia; ^3^ Institute of Molecular Plus Tianjin University Tianjin 300072 China; ^4^ Centre for Atomaterials and Nanomanufacturing (CAN) School of Science RMIT University Melbourne VIC 3000 Australia; ^5^ Department of Chemistry Tsinghua University Beijing 100084 China

**Keywords:** Alloy, Carbon dioxide reduction, Coupled electrocatalysis, Formic acid, Methanol oxidation

## Abstract

The coupling of electrocatalytic CO_2_ reduction (ECR) and methanol oxidation reaction (MOR) presents a promising strategy for simultaneous cogeneration of formic acid (FA) at both cathode and anode. However, sluggish kinetics, low selectivity and efficiency hinder practical application. Herein, we demonstrate an integrated ECR||MOR system employing CuBi cathode and NiCo anode for energy‐efficient FA cogeneration. The CuBi alloy achieves high Faradaic efficiencies (FE > 90%) for FA generation over an extensive potential range (>400 mV), attributed to the accelerated formation of HCOO^*^ intermediates in facilitating FA production. Meanwhile, the NiCo alloy reached a remarkable FE of 97.5% for FA generation at 1.4 V versus reversible hydrogen electrode, benefiting from rapid HCOO^*^ intermediate formation that effectively mitigates CO toxicity. This unique system delivered a current density of 10 mA cm^−2^ at a voltage of 2.07 V, representing a substantial reduction of 320 mV compared to water electrolysis. Across a wide operational voltage window (2.0–2.8 V), the system consistently delivered total Faradaic efficiencies ranging between 189% and 192%, alongside exceptional FA production capacities surpassing 400 g kWh^−1^, which significantly outperformed traditional methods (∼220 g kWh^−1^). This work provides an efficient pathway for low‐energy CO_2_ utilization and sustainable FA production.

## Graphical abstract

An integrated CO_2_ reduction and methanol oxidation system employing CuBi cathode and NiCo anode enables efficient cogeneration of formic acid. The electron‐rich Bi sites accelerate HCOO^*^ formation, while low‐coordination Co atoms enhance methanol oxidation. The coupled system delivers total Faradaic efficiencies of 189%–192% across 2.0–2.8 V, achieving sustainable and energy‐efficient FA production.

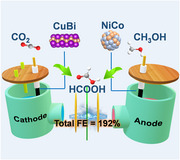

## Introduction

Formic acid (FA) is a critical raw material in the chemical and pharmaceutical industries.^[^ ^1^, ^2^, ^3^ ^]^ Electrocatalytic CO_2_ reduction reaction (ECR) offers a green and sustainable pathway to produce FA, using renewable electricity.^[^ ^4^, ^5^, ^6^, ^7^ ^]^ In a conventional cathodic ECR process, the anode usually simultaneously undergoes an oxygen evolution reaction (OER). However, limited by the extremely slow kinetics of OER, a higher potential (>1.23 V) and greater energy input into the reaction system are required for the smooth progress of ECR, significantly reducing the efficiency of the entire electrocatalytic process.^[^ ^8^, ^9^ ^]^ To overcome these limitations, recent researches has focused on coupling ECR with more favorable anodic reactions. This approach not only reduces the anodic reaction potential and energy consumption but also generates valuable co‐products, thereby further contributing to carbon utilization.^[^ ^10^, ^11^ ^]^ One innovative strategy is coupling ECR with small molecule oxidation. The anodic oxidation reactions with lower onset potentials can be effectively coupled with cathodic ECR, demonstrating dual functionality in ECR and oxidation to value‐added chemicals for energy‐efficient utilization.^[^ ^12^, ^13^, ^14^ ^]^ Among various small molecule alcohols, methanol serves as an upstream substance for FA and has a relatively lower cost (€350 per ton), possessing tremendous potential for electrocatalytic oxidation to produce FA (€492 per ton).^[^ ^15^ ^]^ Therefore, the electrocatalytic methanol oxidation reaction (MOR) and ECR can not only effectively pair but also have significant implications for focusing on the production of FA in both parts, maximizing carbon utilization and improving the overall energy efficiency of the electrocatalytic system.

In the ECR process, since the formation of ^*^CO_2_^−^ requires a very negative redox potential (−1.9 V, relative to the standard hydrogen electrode (SHE)), it is crucial to promote the generation of ^*^CO_2_^−^ by a rational catalyst design, however, the various pathways for the further conversion of *CO_2_^−^ lead to a low product selectivity.^[^ ^16^, ^17^ ^]^ Therefore, the targeted conversion of ^*^CO_2_^−^ is crucial to achieve high product selectivity. The moderate d‐orbital energy level of Cu, which can form effective interactions with CO_2_ molecules and provide the right environment for electron transfer, has made it well‐known in the field of ECR. ^[^ ^18^, ^19^ ^]^ As a result, catalysts based on Cu modification have mushroomed, and defect engineering,^[^ ^20^ ^]^ surface reconstruction,^[^ ^21^ ^]^ heteroatom doping,^[^ ^22^ ^]^ and alloy engineering^[^ ^23^, ^24^, ^25^ ^]^ have been considered as effective strategies to improve the electronic structure of Cu in order to modulate the product type.^[^ ^26^ ^]^ Alloying has attracted much attention because it can improve corrosion resistance and deactivation resistance, provide different functional sites, and optimize the electronic properties of active sites by adjusting the surface composition and structure of metals. ^[^ ^27^, ^28^, ^29^ ^]^ For instance, Xu et al. enhanced the adsorption of Cu on ^*^CO and ^*^OCCO intermediates by introducing Ag through an alloying strategy to achieve the inhibition of C_1_ product outputs.^[^ ^30^ ^]^ A similar strategy was used for the construction of CuBi alloys with different ratios leading to the biased generation of four different products, CO, CH_4_, C_2_H_4_, and HCOOH.^[^ ^31^ ^]^ Recently, Li et al. optimized the adsorption of the reaction intermediate ^*^OCHO by a CuSn alloy obtained through an in situ reconstruction strategy, which promoted the formation of FA (Faradaic efficiency (FE) = 92%) and inhibited the competitive HER.^[^ ^32^ ^]^ Although the alloying strategy to modulate different ECR products is interesting, the moderate FE of a single product is not sufficient for practical applications, and thus the realization of targeted ECR generation still needs to be developed. Furthermore, the generation pathways of different products on alloy catalysts should be elucidated to determine the mechanism of targeted product generation, while better guiding catalyst design rather than trial and error. In addition to Cu, Bi materials are one of the most promising catalytic materials for ECR because they effectively activate CO_2_ molecules and promote ^*^CO_2_^−^ formation.^[^ ^33^, ^34^, ^35^ ^]^ In addition, there is a significant electronegativity difference between Bi (*χ* = 2.02) and Cu (*χ* = 1.90), so that Cu can donate electrons to bismuth in Cu–Bi bimetallic nanostructures, which assists in increasing reactivity of reactants, reduce side reactions, and increase the yield of target products. Therefore, we constructed CuBi alloy catalysts at the cathode for ECR.

On the other hand, the very low potential (0.03 V versus SHE) of methanol oxidation makes it an ideal candidate to replace the OER for reducing the voltage input.^[^ ^36^ ^]^ However, such a low potential makes MOR susceptible to over‐oxidation to worthless CO_2_, reducing the yield of HCOOH.^[^ ^37^ ^]^ Therefore, controlled methanol oxidation for targeted FA production has become a problem to be solved. Typical transition metals (Ni, Co, etc.) and their oxides and hydroxides, have been proven to efficiently catalyze the small molecule alcohols to their corresponding carboxylic acids, inspiring the research direction of constructing Ni and Co‐based catalysts using a straightforward approach for alcohol oxidation as an alternative to OER.^[^ ^38^, ^39^, ^40^ ^]^ Since Ni and Co possess similar crystal structures and have very close lattice parameters, Ni and Co can form solid solution alloys. Co metal can easily adsorb OH^−^ at low potentials, thus promoting the formation of NiOOH^*^ active sites,^[^ ^41^, ^42^ ^]^ and electrophilic oxygen is very favorable for nucleophilic oxidation, and is therefore used in the anodic MOR reaction.

Herein, we propose an advanced integrated electrolyzer configuration that leverages the synergy between CuBi alloy cathodes and NiCo alloy anodes for efficient FA production. A straightforward one‐step electrodeposition was employed to synthesize CuBi alloy cathodes, which displayed exceptional catalytic performance with a sustained 98.0% FE for FA generation over 20 h. This notable performance is attributed to the optimized electronic structure of CuBi alloy, enhancing the selective formation of HCOO^*^ intermediates and suppressing unwanted CO by‐products, as verified by in situ attenuated total reflection infrared spectroscopy (ATR‐IR) spectroscopy and DFT simulations. On the anode side, solvent‐free microwave synthesis produced NiCo alloy electrodes exhibiting remarkable durability and a consistent FE of 97.0% during MOR, driven by efficient HCOO^*^ formation and the avoidance of CO toxicity. Notably, the integrated ECR||MOR electrolyzer demonstrated impressive performance, achieving a current density of 10 mA cm^−2^ at an operating voltage as low as 2.07 V–320 mV below conventional water electrolysis benchmarks. Furthermore, within an operating voltage range from 2.0 to 2.8 V, the system consistently maintained high total FE (189%–192%) and surpassed conventional FA production energy efficiency, achieving over 400 g kWh^−1^.

## Results and Discussion

### Construction of CuBi Alloy Cathode for ECR

**Figure 1:**
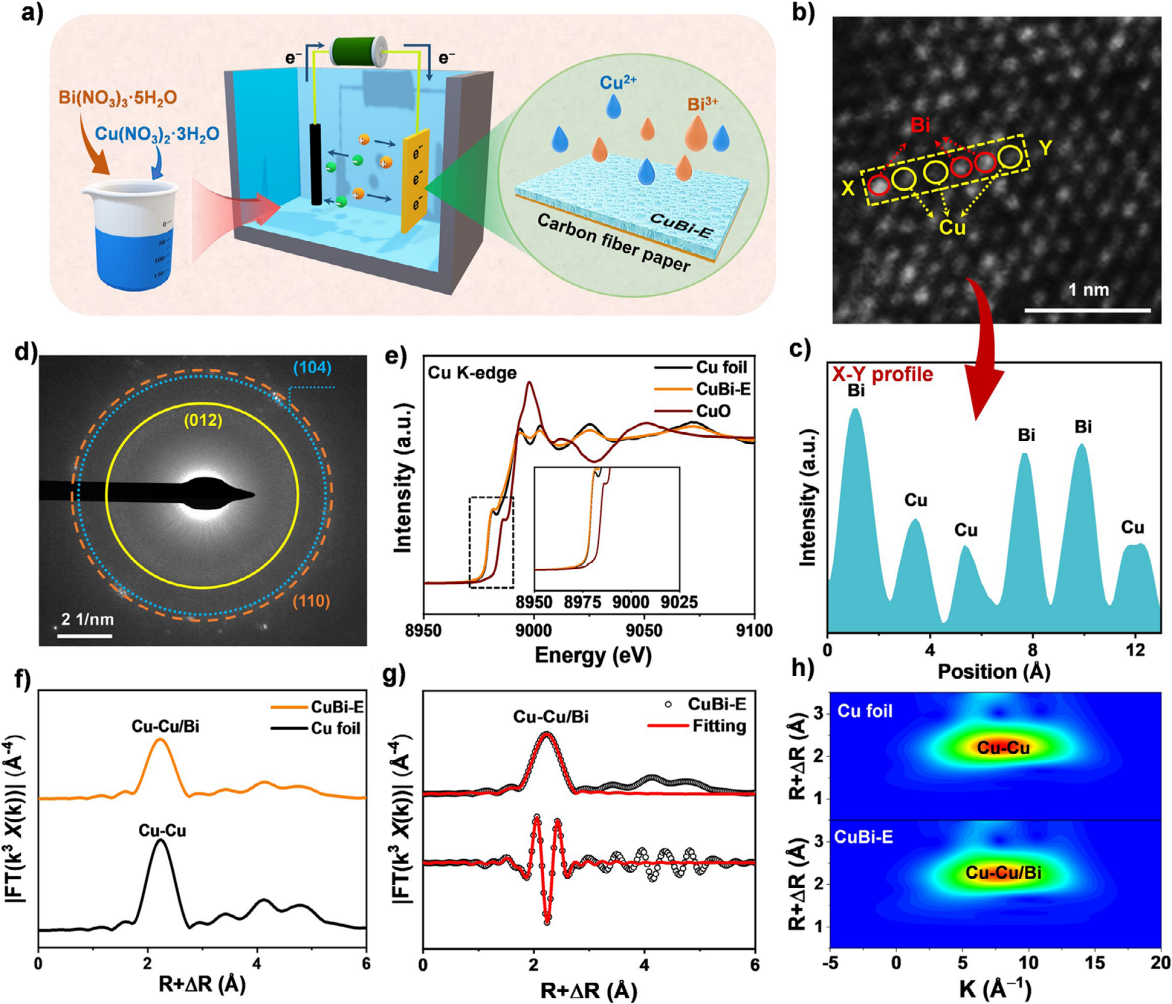
Synthesis, structural and morphological characterizations. a) Schematic illustration of one‐step electrodeposition synthesis of CuBi–E catalyst. b) and c) STEM image and corresponding line profile intensity of CuBi–E catalyst. d) SAED pattern of CuBi‐E catalyst. e) XANES spectra of the normalized Cu K‐edge of CuBi–E catalyst, Cu foil and CuO. f) Fourier transforms of the Cu K–edge of the EXAFS spectra of CuBi–E catalyst and Cu foil, g) fitting result. h) WT EXAFS plots of Cu foil and CuBi–E catalyst.

The bimetallic CuBi alloy (denoted as CuBi–E) was synthesized using a straightforward one‐step electrodeposition strategy (Figure 1a). The specific deposition potential was chosen based on the linear scanning voltammetry (LSV) curve (distinct reduction peak near −0.6 V versus Ag|AgCl) of the electrode substrate in the electrodeposition solution (Figure S1). In contrast, Cu (Cu–E) and Bi (Bi–E) catalysts were also synthesized. Additionally, another bimetallic CuBi alloy (CuBi–S) catalyst was prepared through a conventional solvothermal method. The molar ratio of Bi to Cu was approximately 0.9:1 in both CuBi–E and CuBi–S, as indicated by inductively coupled plasma optical emission spectrometer (ICP‐OES) (Figure S2). The XRD patterns of CuBi–E and Bi–E matched well with the rhombohedral phase of metallic Bi (PDF#85–1329, Figure S3).^[^ ^43^ ^]^ However, the CuBi–E showed a slightly positive shift relative to the standard reference (Figure S4), which could be attributed to the insertion of Cu into the lattice of Bi. In contrast, the CuBi–S catalyst displayed distinct Cu diffraction peaks (PDF#65–9743) indicative of phase segregation, which likely compromises the Bi–Cu synergistic effects and consequently diminishes catalytic efficiency.

X‐ray photoelectron spectroscopy (XPS) was then employed to characterize and analyze the chemical states of elements. The Bi 4f XPS spectra (Figure S5) revealed metallic Bi^0^ states (∼157  and 162 eV) in all catalysts.^[^ ^44^ ^]^ Moreover, the binding energy with 0.68  and 0.37 eV negative shift for CuBi–E (156.58 eV) and CuBi–S (156.89 eV), respectively, originates from electron transfer from less electronegative Cu to Bi, the electron‐rich Bi may favor catalytic activity enhancement.^[^ ^45^ ^]^ Consistently, Cu 2p spectra (Figure S6) showed a 0.09 eV positive shift for CuBi–E (932.48 eV) versus CuBi–S (932.39 eV), indicating weaker Cu–Bi electronic interaction in CuBi–S.^[^ ^46^ ^]^ As shown in Figure S7a, the CuBi–E exhibited a fluffy morphology with distinct spaces**_,_** while the CuBi–S particles showed irregular bulk stacking (Figure S7b). High‐resolution transmission electron microscope (HRTEM) images revealed a visible lattice spacing of approximately 0.327 nm for CuBi–E catalyst, corresponding to the (012) plane of the rhombohedral phase of Bi (Figure S8). Scanning transmission electron microscope (STEM) was then employed to closely analyze the approximate distribution of bimetallic atoms. The line intensity distribution in Figure 1c was derived from the regions labelled in Figure 1b, where Cu atoms had a smaller mass and radius than the Bi atoms and exhibited relatively lower contrast, reflected by the difference in line intensity with Bi.^[^ ^47^ ^]^ It was evident that Cu atoms were entirely located within the Bi lattice and were abundantly distributed. The elemental distributions were further confirmed by energy dispersive spectroscopy (EDS) (Figures S9 and S10), and the results showed that the distributions of Bi and Cu in the CuBi–E catalysts were completely homogeneous. These findings further confirmed that Cu and Bi form a compact alloy phase. Additionally, the selected area electron diffraction (SAED) pattern displayed several distinct diffraction rings, sequentially corresponding to the (012), (104) and (110) planes (Figure 1d) of Bi, perfectly aligning with the strongest diffraction peaks observed in the XRD patterns.

The electronic states and atomic configurations of the CuBi–E catalyst were further investigated using synchrotron X‐ray absorption fine structure (XAFS) spectroscopy. Figure 1e presents the Cu K‐edge X‐ray absorption near‐edge structure (XANES) spectra of CuBi–E compared with commercial Cu foil and CuO references. Notably, the Cu K‐edge absorption threshold of CuBi–E aligns with that of metallic Cu foil, unequivocally confirming the predominant metallic state of Cu species in the catalyst. Further structural insights were obtained through Fourier‐transform analysis of the k^3^‐weighted extended X‐ray absorption fine structure (EXAFS) spectra (Figure 1f,g and Table S1). The Fourier‐transform EXAFS profile of CuBi–E exhibits a prominent peak at 2.2 Å, corresponding to Cu–Cu/Bi bonding interactions. This coordination environment closely resembles the Cu‐Cu metallic bonding observed in Cu foil, with refined fitting parameters revealing a coordination number of 8.4 and a bond distance of 2.54 Å, which is very close to the Cu─Cu bond length of 2.55 Å.^[^ ^48^ ^]^ This indicates that the Bi atom has replaced the Cu atom to form the CuBi alloy phase. Complementary wavelet transform (WT) analysis further corroborates this conclusion, displaying a characteristic intensity maximum at 7.6 Å^−1^ (Figure 1h) that is diagnostic of Cu–Cu/Bi metallic interactions in the alloy system.

**Figure 2:**
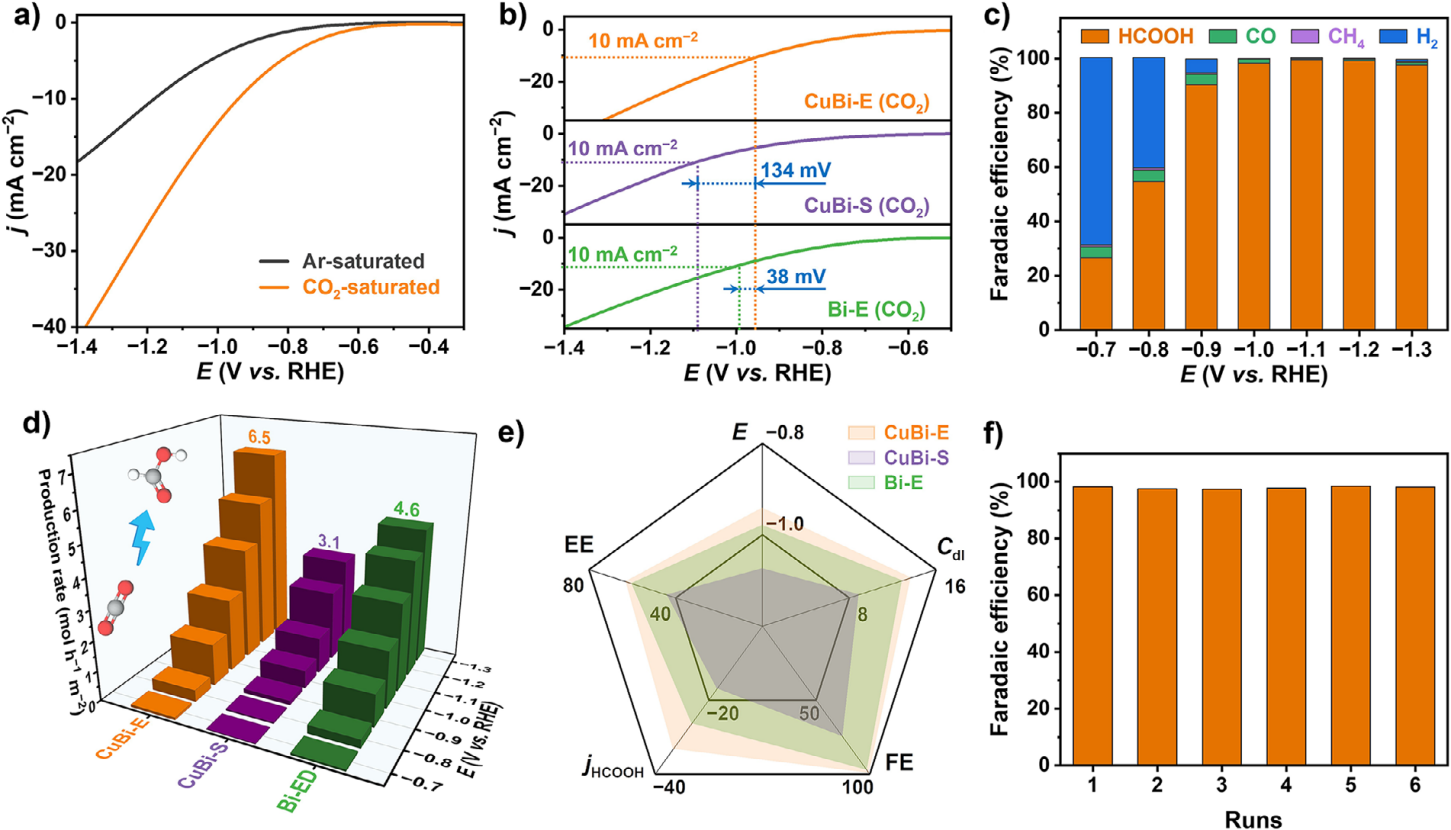
Electrocatalytic performance at the cathode for ECR. a) LSV curves of CuBi–E electrode in 0.1 M KHCO_3_ solution saturated with Ar or CO_2_. b) LSV curves of CuBi–E, CuBi–S and Bi–E electrodes in CO_2_‐saturated 0.1 M KHCO_3_ solution. c) FE of ECR products at different potentials for CuBi–E electrode. d) FA production rates of CuBi–E, CuBi–S and Bi–E electrodes at different potentials. e) Comparison of ECR performance metrics for CuBi–E, CuBi–S and Bi–E electrodes, including reaction potential (*E*), double‐layer capacitance (*C*_dl_), FE, FA partial current density (*j*_HCOOH_) and energy efficiency (EE). f) Cyclic stability of CuBi–E.

The ECR performance of CuBi–E, CuBi–S, Cu–E and Bi–E electrodes was investigated in the CO_2_‐saturated 0.1 M KHCO_3_ solution. CuBi–E, CuBi–S, and Bi–E exhibited a higher current response than in argon‐saturated conditions (Figures 2a and S11), suggesting a higher sensitivity of ECR relative to the hydrogen evolution reaction (HER). CuBi–E electrodes achieved potential reduction of 134 and 38 mV at a current density of −10 mA cm^−2^ compared to CuBi–S and Bi–E, respectively, suggesting that the highest ECR activity of CuBi–E electrodes (Figure 2b). Moreover, the current density of CuBi–E electrode remained the highest in the range of − 0.7 to − 1.3 V versus RHE (Figures S12–S13). Electrochemical impedance spectroscopy (EIS) results presented in Figure S14 showed that CuBi–E electrode exhibited a charge transfer resistance (*R*_ct_) of approximately 25.1 Ω in the ECR system, significantly lower than that of the CuBi–S electrode (127.4 Ω) and Bi–E electrode (39.3 Ω). The fast charge transfer favored the ECR reaction. The *C*_dl_ values of CuBi–E, CuBi–S and Bi–E electrodes were found to be 13.7, 8.9 and 12.9 mF cm^−2^, respectively (Figure S15). The highest *C*_dl_ of CuBi–E electrode indicated its superior capability to facilitate mass transfer and provide more active sites, which was advantageous for the efficient conversion of CO_2_. Furthermore, the electrochemically active surface area (ECSA) normalized LSV curve (Figure S16) confirms that CuBi–E exhibits the highest performance, indicating superior intrinsic activity compared to CuBi–S and Bi–E, which in turn accounts for its outstanding apparent ECR activity.

Then, constant potential electrolysis was employed to record the chronoamperometric (CA) curves of CuBi–E, CuBi–S, Cu–E and Bi–E electrodes at various potentials (Figure S17). At all potentials from − 0.7 to − 1.3 V, the CuBi–E electrode exhibited the highest current density. Notably, CuBi–E electrode maintained an exceptionally stable current density at each potential, demonstrating considerable ECR stability. The liquid and gaseous products were detected by high‐performance liquid chromatography and gas chromatography, respectively (Figures S18 and S19). Potential‐dependent FE of FA showed an increase from 26.7% at − 0.7 V versus RHE to 90.4% at − 0.9 V versus RHE (Figure 2c). In addition, the FE consistently remains above 97.0% as the potential was further increased, reaching a peak of 99.6% at − 1.1 V versus RHE. In contrast, the FA generation performance of Cu‐E electrode is far inferior to that of CuBi–E electrode (Figure S20a), CuBi–S achieving a peak FE of only 74.9% at − 1.2 V versus RHE (Figure S20b). In addition, the FE of FA over Bi–E electrode in the − 1.1 to − 1.3 V range is inferior to that of the CuBi–E electrode (Figure S20c). The FA partial current density clearly demonstrated the superior performance of the CuBi–E electrode in FA production (Figure S21). Furthermore, the Tafel slope for CuBi–E electrode was 175 mV dec^−1^, lower than that of CuBi–S electrode (209 mV dec^−1^) and Bi–E (179 mV dec^−1^) electrode, illustrating the exceptional kinetics of CuBi–E electrode in converting CO_2_ to FA (Figure S22). The energy efficiency represents the efficiency of converting electrical energy into tangible FA production.^[^ ^49^ ^]^ As illustrated in Figure 2d and Figure S23, CuBi–E electrode exhibited the highest energy efficiency across the tested potential window of 600 mV, compared to CuBi–S electrode and Bi–E electrode, reaching a maximum energy efficiency of nearly 65% around − 1.0 V versus RHE, which implied that the CuBi–E electrode can effectively produce a significant amount of FA with lower energy consumption.

Integrating the above ECR performance metrics, a systematic comparison of CuBi–E, CuBi–S, Cu–E and Bi–E electrodes demonstrated a comprehensive superiority of CuBi–E electrode in ECR performance over CuBi–S, Bi–E, and Cu–E electrodes (Figure 2e). Combined with the previous characterization results, it can be understood that the optimized electronic structure due to the electron migration between Cu and Bi facilitates the ECR. The unique electronic coupling between Cu and Bi in the CuBi–E catalyst is beneficial in mitigating the risk of catalyst component loss and deactivation, as evidenced by the quantified loss rates of Cu and Bi after 6 hours of electrolysis (Figure S24). To evaluate the cyclic stability of the CuBi–E electrode in ECR, six repeated runs were conducted at a potential of − 1.0 V versus RHE, as illustrated in Figure 2f. The FE for FA was able to be maintained consistently around 98.0% across all cycles, with no discernible trend toward degradation. After the cyclic operations, the LSV characteristic curve remained nearly identical to those observed before the reaction, further underscoring the excellent stability of the CuBi–E (Figure S25). The XRD and SEM results indicated that the phase structure and morphology of the CuBi–E catalyst after the reaction are well‐maintained (Figures S26 and S27).

### Study of the ECR Reaction Mechanism Using CuBi–E Catalyst

**Figure 3:**
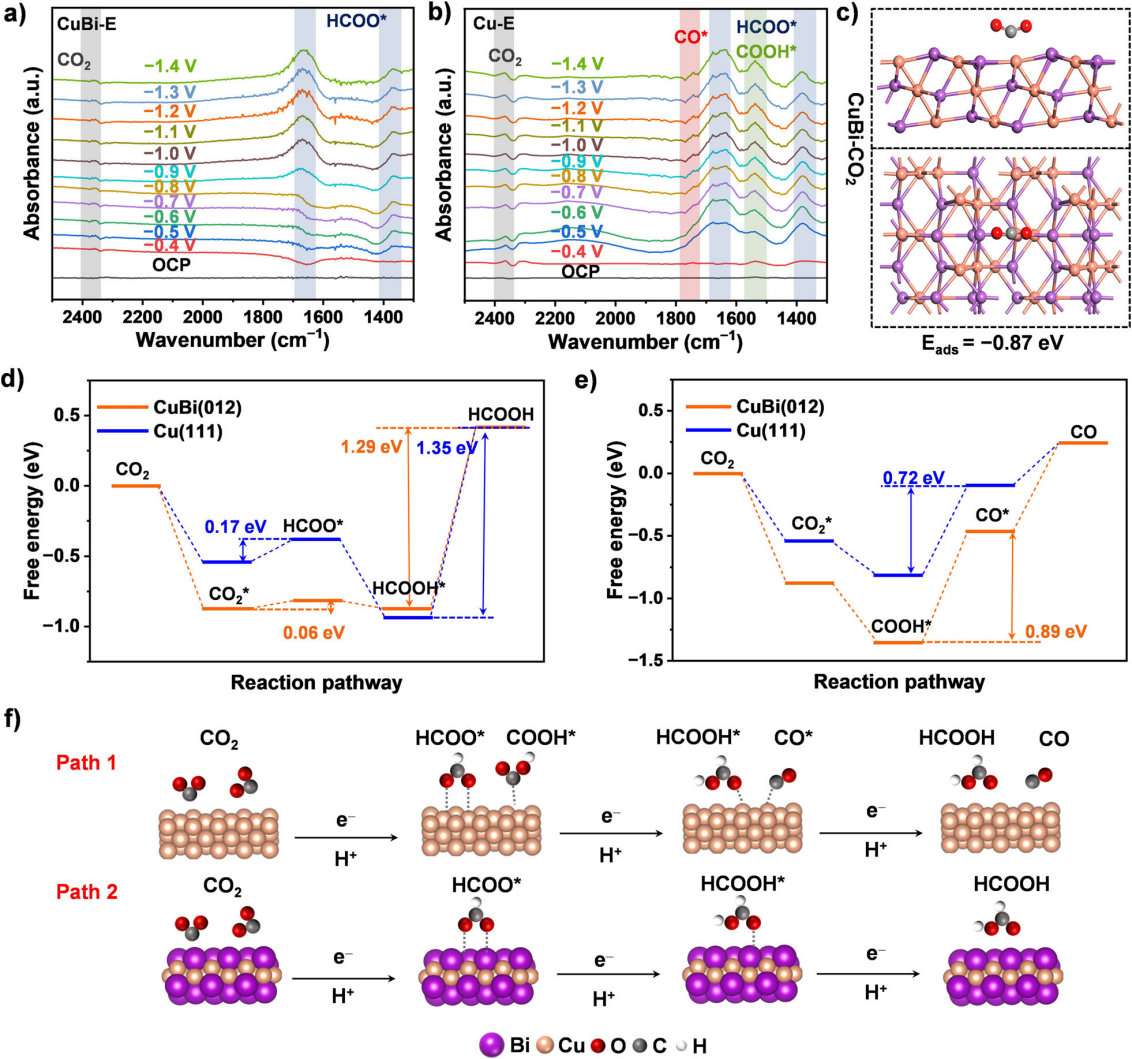
In situ ATR‐IR and theoretical calculations. In situ ATR‐IR spectra for ECR on a) CuBi–E electrode and b) Cu–E electrode. c) CO_2_ adsorption model on CuBi. d) The Gibbs free energy of the HCOOH pathway on Cu(111) and CuBi(012). e) The free energy of the CO pathway on Cu and CuBi alloy. f) Schematic diagram of two paths for electrocatalytic conversion of CO_2_.

To investigate the reaction pathways during the ECR at the molecular level, in situ attenuated total reflection infrared spectroscopy (ATR‐IR) measurements were performed in combination with electrochemical techniques, collecting spectra at 0.1 V potential intervals. As depicted in Figure 3a, the CuBi–E catalyst with optimal ECR performance exhibited characteristic vibrational signatures at approximately 1390 and 1650 cm^−1^ when polarized from − 0.4 V versus RHE, corresponding to HCOO^*^ intermediate formation. Notably, these FA‐related signals intensified progressively with increasing applied potentials, while the CO_2_ substrate vibration at ∼2350 cm^−1^ remained observably. It was noted that CO_2_ signal on the Cu–E catalyst was stronger than on the CuBi–E catalyst, indicating that CO_2_ reacts slowly on Cu–E and hydrogenation of CO_2_ does not occur easily. The absence of other discernible intermediate peaks suggests a singular reaction pathway through the HCOO^*^ intermediate for FA production, thus exhibiting high FA selectivity.^[^ ^50^ ^]^ However, in the monometallic Cu used as a control catalyst, upon polarization from − 0.6 V versus RHE, distinct vibrational features emerged at ∼1550 cm^−1^ (COOH^*^) and ∼1750 cm^−1^ (CO^*^),^[^ ^51^ ^]^ indicative of competing reaction pathways involving CO‐related intermediates. (Figure 3b). These CO intermediates strongly adsorbed onto the catalyst surface, which not only facilitated the production of CO gas during the ECR process but also led to catalyst poisoning by the adsorbed CO^*^, hindering the reaction and resulting in decreased electrocatalytic performance and poorer product selectivity. Therefore, the CuBi–E electrode demonstrated excellent catalytic performance and FA selectivity by regulating the reaction intermediates. To elucidate the intrinsic mechanism underlying the enhanced FA formation from CO_2_ reduction over CuBi alloy, density functional theory (DFT) calculations were carried out. Surface models of Cu (111) and CuBi (012) were constructed to investigate their CO_2_ reduction pathways (Figure 3c). The DFT calculations reveal that CO_2_ adsorption exhibits spontaneous behavior on both surfaces, with more favorable adsorption energy on CuBi (012) (−0.87 eV) compared to Cu (111) (−0.55 eV), indicating stronger CO_2_ activation capability of the CuBi alloy. For the FA formation pathway (Figures 3d and S28), the protonation of adsorbed CO_2_^*^ to form HCOO^*^ intermediate presents an energy barrier of 0.17 eV on Cu (111), which is significantly reduced to 0.06 eV on CuBi (012), demonstrating the catalytic advantage of the alloy surface in facilitating this critical step. The subsequent desorption of HCOOH* constitutes the rate‐determining step (RDS) for HCOOH production, where CuBi shows a lower activation barrier (1.29 eV) compared to pure Cu (1.35 eV), further confirming the thermodynamic preference for HCOOH generation on the alloy surface. In contrast, the CO formation pathway (Figures 3e and S29) reveals different mechanistic behavior. While the initial formation of COOH^*^ from CO_2_^*^ is more favorable on CuBi (012), the subsequent CO^*^ generation step becomes the RDS with a higher energy barrier (0.89 eV) compared to pure Cu (0.72 eV). This energy disparity explains the suppressed CO production and preferential HCOOH selectivity observed on CuBi alloys. The above results indicate that CuBi (100) is thermodynamically and kinetically more favorable for HCOOH generation, which is consistent with the experimental results. Overall, CO_2_ is more likely to generate FA on CuBi via path 1, while on Cu it is more likely to generate CO via path 2 (Figure 3f).

### Construction of NiCo Alloy for Anode MOR

**Figure 4:**
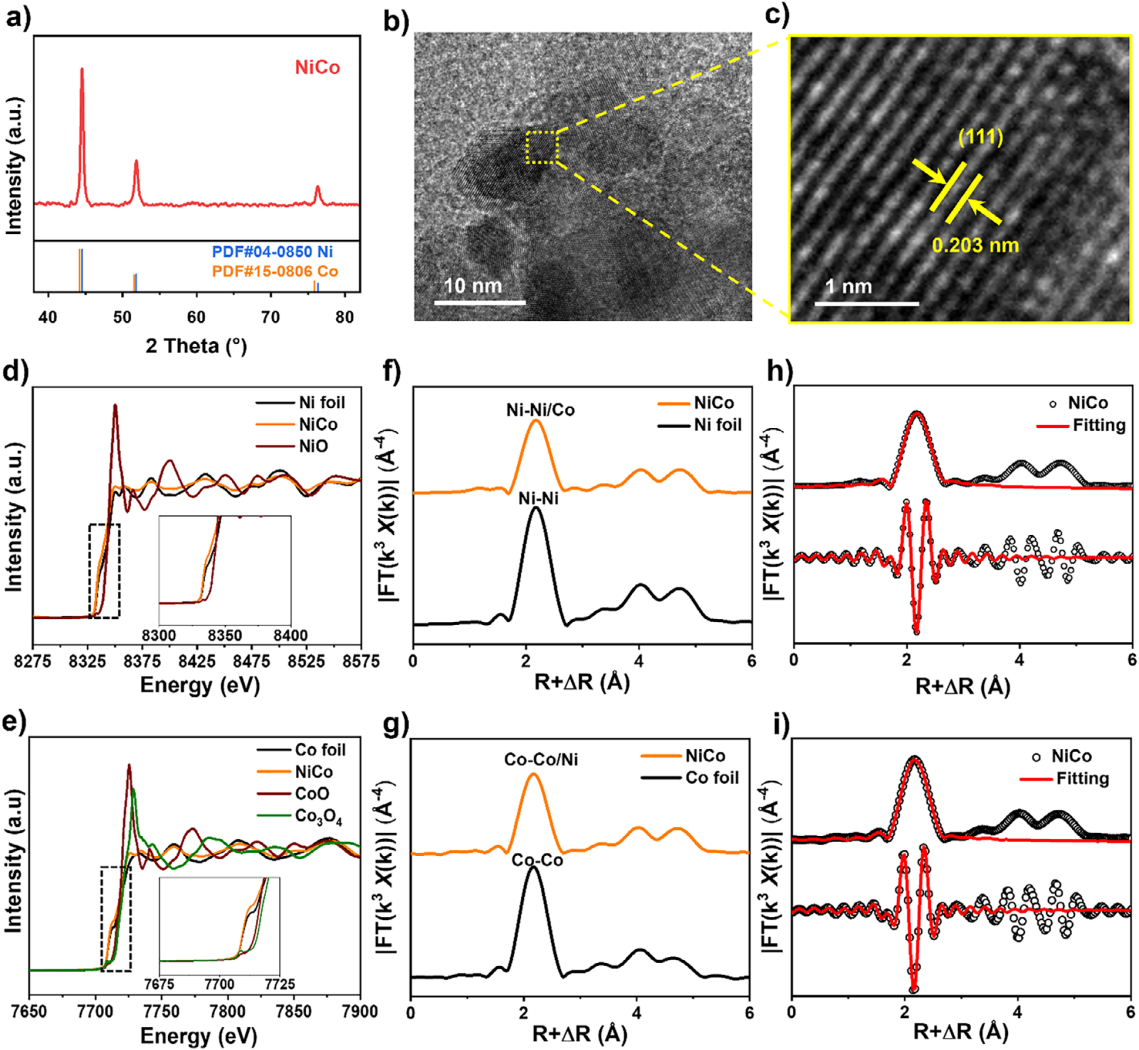
Structural and morphological characterizations of NiCo alloy. a) XRD pattern of NiCo alloy. b) HRTEM and c) corresponding magnified images. d) XANES spectra of the normalized Ni K‐edge of NiCo alloy, Ni foil, e) Co K‐edge of NiCo alloy, Co foil,CoO and Co_3_O_4_. f) Fourier transforms of the Ni K‐edge of the EXAFS spectra of NiCo alloy and Ni foil, g) Co K‐edge of the EXAFS spectra of NiCo alloy and Co foil. Fitting result of Fourier transforms of the h) Ni K‐edge, i) Co K‐edge of the EXAFS spectra of NiCo catalyst.

For the anode, a straightforward one‐step microwave‐assisted synthesis method (Figure S30) was utilized to construct a bimetallic NiCo alloy catalyst for MOR. The metallic content was precisely quantified using ICP‐OES (Figure S31), the molar ratio of Ni to Co was approximately 1.3:1, which was close to the ratio of precursors. The crystal structure characteristics of the NiCo catalyst were elucidated using XRD (Figure 4a). Reference to the standard diffraction patterns for Ni (PDF#04–0850) and Co (PDF#15–0806), it revealed the presence of a bimetallic state of cubic phase in NiCo alloy catalyst. Then, HRTEM images revealed a plane spacing of 0.203 nm, corresponding to the most exposed plane of the NiCo (111) (Figure 4b,c). The SAED pattern in Figure S32 showed the brightest (111) and (200) diffraction rings, further proving the metallic state of NiCo alloy. The elemental line intensity analysis (Figure S33a) of the marked area in Figure S33b indicated that strong signals of both Ni and Co, with the line intensity peak and valley distribution trends being generally consistent, indicating the generation of NiCo alloy phase, and the EDS in the corresponding region also exhibits a uniform distribution of Ni and Co (Figures S33c, S33d), which is further evidence of the generation of the NiCo alloy phase.

XAFS measurements were conducted to probe the electronic states and atomic configurations of Ni and Co in the NiCo catalyst. As shown in Figure 4d, the Ni K‐edge XANES spectra exhibit near‐edge absorption features nearly identical to metallic Ni foil. Similarly, the Co K‐edge XANES profile (Figure 4e) demonstrates close alignment with metallic Co foil, confirming the metallic nature of both Ni and Co in the catalyst. Further structural analysis through Fourier‐transformed EXAFS at the Ni K‐edge (Figure 4f) reveals that the NiCo catalyst maintains coordination characteristics similar to pure Ni foil, with comparable peak positions and intensities in the radial distribution function. Similarly, the Fourier transform EXAFS spectra of the Co K‐edge also prove that Co is in a metallic state (Figure 4g). The fitting results show (Figure 4h, i and Tables S1,S2) that the coordination number (CN) of Co in the NiCo alloy is 5.9, which is much lower than that of Co foil, suggesting that there are more unsaturated Co atoms in the NiCo alloy, which may allow for a higher chemical activity. In addition, the Co─Co/Ni bond and Ni─Ni/Co bond lengths of 2.48 and 2.49 Å are extremely close to the Co─Co bond length of 2.49 Å for Co foils, which suggests that Ni replaces Co to form Ni─Co bonds. Furthermore, WT ‐EXAFS (Figure S34) was used to investigate the metal‐neighboring properties, both Ni and Co have only Ni─Ni/Co coordination and Co─Co/Ni signals, indicating the successful synthesis of NiCo alloy catalysts in the metallic state. Based on the above XAFS results, it can be concluded that the formation of significant Ni‐Co direct coordination within NiCo catalyst further confirms the formation of alloy structure.

**Figure 5:**
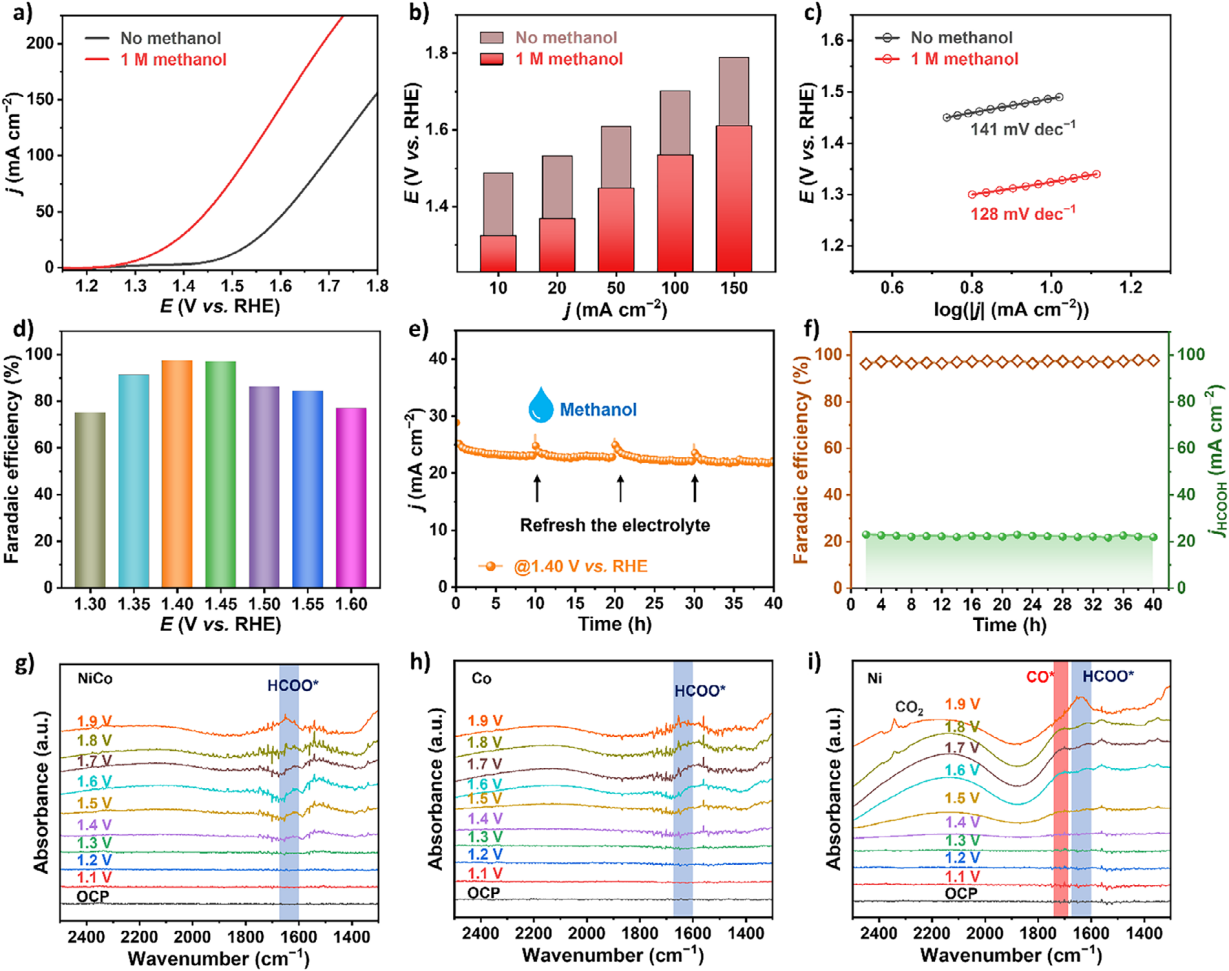
Electrocatalytic performance at anode toward MOR on NiCo electrode. a) LSV curves in 1 M KOH solution with and without 1 M methanol. b) Comparison of the potentials required to achieve different current densities in KOH solution with and without methanol. c) Tafel slopes in KOH solution with and without methanol. d) FE of methanol oxidation to FA at different potentials. e) Long‐term CA test curves for MOR at constant potential. f) FE and FA partial current density over time during the CA test. Potential‐dependent in situ ATR‐IR spectra of g) NiCo, h) Co, i) Ni.

The electrocatalytic performance of the NiCo catalyst for MOR was investigated. Figure 5a shows the LSV curves in 1 M KOH solution before and after the addition of 1 M methanol. A distinct enhancement in current density was observed after the addition of 1 M methanol, indicating that MOR is more favorable than OER. At current densities up to 10 mA cm^−2^, the potential was 1.487 V without methanol and 1.324 V with methanol, a decrease of 163 mV. A similar decrease of about 160 mV was observed at current densities of 20, 50, 100, and 150 mA cm^−2^ (Figure 5b). This suggests that MOR is thermodynamically more favorable than OER. Derived Tafel plots showed a slope of MOR with 128 mV dec^−1^, lower than that of the OER (141 mV dec^−1^), indicating faster catalytic reaction kinetics (Figure 5c). Furthermore, EIS results depicted in Figure S35 demonstrated that the *R*_ct_ of the reaction system significantly decreased from approximately 21.4 Ω to about 10.2 Ω after the addition of methanol. ECSA measurements revealed an exceptionally expansive active area for NiCo, with a *C*_dl_ value of 0.207 F cm^−2^ (Figure S36). These outcomes collectively suggested a pronounced capacity of NiCo electrode for enhancing mass transport and augmenting the availability of active sites during the MOR process. The performance of NiCo catalyst for MOR was evaluated at different potentials, FA has been identified as the primary product. As depicted in Figure 5d, the FE of FA exhibited a volcanic trend with the rise in potential, reaching over 80% within the range of 1.35 to 1.55 V versus RHE. Notably, the peak efficiencies of 97.5% and 97.2% were achieved at 1.40 and 1.45 V versus RHE, respectively, indicating a remarkably high selectivity for the conversion of methanol to FA. The low potential was not sufficient to drive the methanol oxidation reaction, hence the low FE, while at more positive potentials, the FE decreased due to the effect of OER.

Furthermore, during prolonged constant potential electrolysis at 1.40 V versus RHE shown in Figure 5e, the current density was able to stabilize around 23 mA cm^−2^. Following a refreshment of the electrolyte at the 10 h mark, the current‐time profile remained consistent with the previous phase, implying the long‐term stability of NiCo alloy in the MOR system. Periodic sampling every 2 h to ascertain the concentration of FA (Figure S37) revealed the stable generation of FA, as evidenced by the Faradaic efficiency (FE) consistently exceeding 97% and the partial current density for FA remaining above 22 mA cm^−2^ throughout the experiment (Figure 5f). Post long‐term electrolysis, the LSV curves in Figure S38 indicated that the potential required at a current density of 100 mA cm^−2^ increased by merely 20 mV. The ICP results showed that the concentrations of Ni and Co in the electrolyte were below the detection limit. The ICP results of the electrolyte after the reaction showed that the no loss of Ni and Co (Figure S39), and the SEM and XRD indicated that the morphology and phase structure of the catalyst is well‐contained remained stable after the reaction MOR (Figures S40 and S41), demonstrating the good catalytic stability of NiCo alloy. To further investigate the MOR mechanism on the NiCo alloy, electrochemical in situ ATR‐IR was employed. As shown in Figures 5g–i, a characteristic peak at ∼1650 cm^−1^, attributed to the HCOO intermediate, was observed on all samples. Notably, this signal appeared at a lower potential on NiCo (1.4 V versus RHE) compared to Co (1.5 V) and Ni (1.6 V), highlighting the superior activity of the NiCo alloy for facilitating HCOO^*^ formation.^[^ ^52^, ^53^ ^]^ For the Ni catalyst, extra characteristic peaks of the CO^*^ intermediate appeared at around 1660 cm^−1^ and CO_2_ at 2350 cm^−1^ were observed as the applied potential increased. This indicated that the Ni catalyst suffered from poisoning by CO^*^ during the MOR process, and it also promoted over‐oxidation of methanol to CO_2_, thereby reducing the FA generation. Therefore, the NiCo alloy not only reduced the reaction potential and enhanced the overall MOR performance but also steered the reaction pathway toward nearly complete and selective FA formation. These comprehensive findings underscore the outstanding electrocatalytic activity of the NiCo alloy, establishing it as an ideal anodic counterpart for coupling with cathodic ECR. This effectively completes the design of a fully integrated and synchronous FA cogeneration system.

### Electrocatalytic Performance of Coupled Reaction System

**Figure 6:**
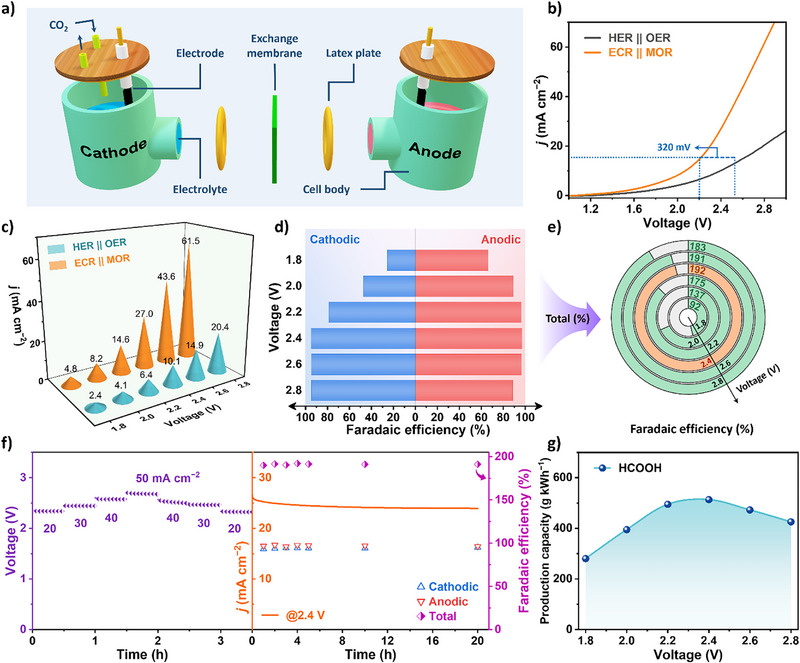
Electrocatalytic performance of CuBi alloy for ECR coupled with NiCo alloy for MOR in synchronous reaction system. a) Schematic diagram of the coupled electrolysis cell. b) LSV curves for the HER||OER and ECR||MOR systems. c) Current densities at different cell voltages for the HER||OER and ECR||MOR systems. d) Cathodic and anodic and e) total Faradaic efficiency for FA in the ECR||MOR system at different applied voltages. f) Continuous constant current and constant potential processes in coupled systems, and cathodic, anodic and total Faradaic efficiency over time. g) The relationship between FA production capacity and applied voltage.

Inspired by the electrocatalytic performance of CuBi alloy (CuBi–E) for the cathodic ECR and NiCo alloy for the anodic MOR, we constructed a two‐electrode electrolyzer for the simultaneous ECR and MOR (Figure 6a). As shown in Figure S42, based on the HER||OER system and substituting only the cathodic reaction with ECR or the anodic reaction with MOR, the constructed ECR||OER and HER||MOR systems were both able to promote the synchronous reactions, with the MOR having a more significant effect. Within the ECR||MOR system, an impressive current density of 10 mA cm^−2^ was achieved at a mere cell voltage of 2.07 V, which was 320 mV lower than that of the HER||OER system (Figure 6b). Moreover, as the voltage increased, this differential trend became even more pronounced. Between 1.8 to 2.8 V of cell voltage, the current density attained by the ECR||MOR system consistently exceeded more than twice that of the HER||OER system (Figure 6c). In Figure S43, stable CA curves were presented within the coupled system, where cathodic and anodic liquid samples were analyzed post‐reaction to calculate the FE of FA. It was observed that with the increase of voltage, the anodic FE rose swiftly, surpassing 90% beyond 2.0 V, indicating that MOR significantly propelled the overall reaction forward (Figure 6d). Subsequently, the cathodic ECR achieved over 80% FE after 2.2 V, with both sides reaching peak efficiencies at 2.4 V (94.8% and 97.1%, respectively). The sum of FE for FA production exceeded 175% between 2.2 to 2.8 V, astonishingly hitting 192% at 2.4 V (Figure 6e). This highlighted the exceptional efficiency of ECR||MOR system in the simultaneous process. To verify the durability of ECR||MOR system, preliminary steps included gradually increasing the current density from 20 to 50 mA cm^−2^ (The critical region for the transition from kinetic control to mass transfer control.) and then reducing it to 20 mA cm^−2^ to alter its dynamic operating environment. Subsequently, CA curves were recorded while maintaining a voltage of 2.4 V for 20 hours of electrolysis (Figure 6f). The ECR||MOR system maintained an extremely stable operational state within 20 h, with the current density sustained above 20 mA cm^−2^. FA concentration was quantitatively analyzed at 1, 2, 3, 4, 5, 10 and 20 h, demonstrating a steady increase in FA production (Figure S44). Throughout the process, the total FA remained consistently between 189% and 192%, showcasing exceptional stability under these conditions and affirming its capability for long‐term operation. After the long‐term stability test, the LSV test was conducted again, revealing only a slight degradation in the performance of the ECR||MOR system (Figure S45). By calculating the total production of FA, the actual production capacity of the ECR||MOR system in relation to the cell voltage was depicted in Figure 6g. Over a broad working voltage range of 2.0 to 2.8 V, the system achieved a production capacity of more than 400 g kWh^−1^. Therefore, the paired ECR||MOR system effectively reduced the electrical input requirements for the production of FA, making it a highly efficient and sustainable option for co‐converting CO_2_ and methanol into valuable chemical products.

## Conclusion

In summary, we have constructed an integrated electrolyzer system featuring synergistic CuBi alloy cathodes and NiCo alloy anodes for efficient formic acid (FA) production. The CuBi cathode, fabricated via simple one‐step electrodeposition, demonstrated 98.0% FE for FA generation over 20 h. In situ ATR‐IR and DFT calculations demonstrate that the optimized electronic structure CuBi alloy not only activates CO_2_ more easily and produces HCOO^*^ faster, but also avoids competing reaction pathways involving CO^*^ related intermediates, making the high coverage of the intermediate HCOO^*^ more favorable for the generation of FA. In addition, CuBi forms a compact alloy phase that greatly limits its metal spillage, resulting in superior durability. NiCo alloy anodes containing more unsaturated Co atoms synthesized through solvent‐free microwave methods showed exceptional stability with 97.0% FE of FA during MOR. The in situ ATR‐IR results demonstrate that NiCo alloys exhibit superior activity in promoting the formation of HCOO^*^, which favors the formation of FA, and also hinders the formation of CO^*^, which avoids the over‐oxidation of methanol and directs the reaction pathway toward near‐complete and selective formation of FA. The coupled ECR||MOR system achieved a low operating voltage of 2.07 V at 10 mA cm^−2^ – 320 mV below conventional water electrolysis – while maintaining high total FE (189%–192%) across 2.0–2.8 V. Remarkably, the system attained high energy efficiency exceeding 400 g kWh^−1^ for FA production, significantly surpassing conventional FA production benchmarks.

## Supporting Information

The Authors have cited additional references within the Supporting Information.^[^ ^54^, ^55^, ^56^, ^57^, ^58^ ^]^

## Conflict of Interests

The authors declare no conflict of interest.

## Supporting information



Supporting Information

## Data Availability

The data that support the findings of this study are available from the corresponding author upon reasonable request.
