# Supplementary material for: Harnessing Electrocatalytic Coupling of Carbon Dioxide and Methanol for High‐Efficiency Formic Acid Production
Source: Angew Chem Int Ed Engl. 2025 Sep 6;64(43):e202512078. doi: 10.1002/anie.202512078 (PMC12535387; doi:10.1002/anie.202512078)
Supplement: Supplementary file 1 — Supporting Information [file ANIE-64-e202512078-s001.docx]

**Supporting Information**

**Harnessing Electrocatalytic Coupling of Carbon Dioxide and Methanol for High-Efficiency Formic Acid Production**

Zhikeng Zheng^a, #^, Xiaobo Zheng^b, #^, Ligang Wang^c, #^, Huiming Wen^a^, Ke Li^a^, Zhenhao Xu^a^, Yameng Fan^d^, Peng Li^d^, Suyu Zhang^a^, Bin Liu^a^, Dingsheng Wang^e^, Kai Yan^a,^* and Guoxiu Wang^b,^*

^a^School of Environmental Science and Engineering, Sun Yat-sen University, Guangzhou 510275, China

^b^Center for Clean Energy Technology, School of Mathematical and Physical Sciences, Faculty of Science, University of Technology Sydney, Sydney, New South Wales 2007, Australia

^c^Institute of Molecular Plus, Tianjin University, Tianjin 300072, China

^d^Centre for Atomaterials and Nanomanufacturing (CAN), School of Science, RMIT University, Melbourne, VIC 3000, Australia

^e^Department of Chemistry, Tsinghua University, Beijing 100084, China

*Corresponding authors Email address: yank9@mail.sysu.edu.cn (Kai Yan); Guoxiu.Wang@uts.edu.au (Guoxiu Wang)

^#^These authors contributed equally to this work.

**Experimental section**

**Chemicals**

All chemicals were used without further purification. Bismuth nitrate pentahydrate (Bi(NO_3_)_3_·5H_2_O, 99%), copper nitrate trihydrate (Cu(NO_3_)_2_·3H_2_O, 99%), citric acid trisodium salt (Na_3_C_6_H_5_O_7_, 98%), urea (CO(NH_2_)_2_, 99%), cobalt(II) acetylacetonate (C_10_H_14_O_4_Co, Co(acac)_2_, 97%), potassium hydroxide (KOH, 95%) and ethanol (C_2_H_6_O, 99.7%) were procured from Macklin. Nitric acid (HNO_3_, 65~68%) was acquired from Guangzhou Chemical Reagent Factory. Ethylene glycol (C_2_H_6_O_2_, >99%), nickel(II) acetylacetonate (C_10_H_14_O_4_Ni, Ni(acac)_2_, 95%), potassium bicarbonate (KHCO_3_, 99.5%) and methanol (CH_3_OH, ≥99.5%) were purchased from Aladdin. All aqueous solutions were prepared using ultrapure water.

**Synthesis of CuBi-E, Cu-E and Bi-E**

The synthesis of CuBi-E was accomplished through a facile one-step electrodeposition method. A homogeneous aqueous solution of 40 mL was prepared containing 0.01 M Bi(NO_3_)_3_·5H_2_O, 0.01 M Cu(NO_3_)_2_·3H_2_O, 0.1 M CO(NH_2_)_2_, 0.1 M Na_3_C_6_H_5_O_7_, and 0.1 M HNO_3_. This prepared solution served as the electrolyte for electrodeposition conducted within a conventional three-electrode system. Carbon fiber paper was employed as the electrode substrate, with an Ag|AgCl electrode and a graphite rod serving as the reference and counter electrodes, respectively. The electrochemical workstation was set to maintain a constant potential of −0.6 V *vs.* Ag|AgCl, and the electrodeposition was carried out for 15 min. Upon completion of the electrodeposition, the resultant electrode was rinsed with ultrapure water to remove soluble impurities, thereby obtaining a catalyst composed of Bi and Cu, denoted as CuBi-E. The synthesis procedure for Cu-E and Bi-E was identical to that described above, with the only difference being the omission of Cu(NO_3_)_2_·3H_2_O or Bi(NO_3_)_3_·5H_2_O from the electrodeposition solution.

**Synthesis of CuBi-S**

The synthesis of CuBi-S was carried out using a solvothermal method. In the typical synthesis process, 0.01 M Bi(NO_3_)_3_·5H_2_O, 0.01 M Cu(NO_3_)_2_·3H_2_O, and 0.1 M CO(NH_2_)_2_ were added to 50 mL of e C_2_H_6_O_2_. The mixture was stirred until it became completely homogeneous. Subsequently, the solution was transferred into a high-pressure autoclave. The solvothermal process was maintained for 12 h at a constant temperature of 180 ℃. After cooling to room temperature, the solid product was separated by centrifugation, followed by washing and drying. The resultant sample was denoted as CuBi-S.

**Synthesis of NiCo alloy**

The synthesis of NiCo alloy was directly achieved using a one-step microwave-assisted method. In the typical synthesis process, equimolar amounts (0.1 mol each) of Ni(acac)_2_ and Co(acac)_2_ were mixed and thoroughly ground together to form a mixture with uniform color and texture. This mixture was then transferred to a microwave furnace. The process was carried out under a H_2_/Ar atmosphere. Initially, the mixture was heated at a constant microwave power of 280 W to reach 200 ℃ and maintained at this temperature for 30 min. Subsequently, the power was abruptly increased to 1300 W to rapidly heat the furnace interior to 500 ℃ and this temperature was held for 15 min. After the microwave-assisted heating was completed, the sample was allowed to cool naturally to room temperature, and the final product was denoted as NiCo alloy.

**Characterization**

X-ray diffraction (XRD) was conducted using a Rigaku Ultima IV diffractometer under conditions of 40 kV and 40 mA using Cu K_α_ radiation. Inductively coupled plasma optical emission spectrometry (ICP-OES) was performed using an Agilent 720ES instrument. X-ray photoelectron spectroscopy (XPS) measurements were taken with a Thermo Scientific spectrometer with an Al K_α_ radiation source operating at 12 kV. Field emission scanning electron microscopy (SEM) images were captured using a Zeiss Sigma 500. Transmission electron microscopy (TEM), selected area electron diffraction (SAED), and scanning transmission electron microscopy (STEM) images were recorded on an FEI F200X microscope, which was equipped with an energy dispersive X-ray spectroscopy (EDS) detector.

**Electrochemical measurements**

All electrochemical experiments were conducted at room temperature using a CHI760E electrochemical workstation. For the independent study of cathodic or anodic reaction system, a three-electrode electrochemical system was employed, with a graphite rod serving as the counter electrode and carbon fiber paper loaded with catalysts acting as the working electrode. Specifically, carbon fiber papers loaded with CuBi-E or Bi-E samples, obtained via the electrodeposition process, were directly used as working electrodes. In contrast, CuBi-S synthesized via the solvothermal method and NiCo alloy synthesized via the microwave method were prepared by dispersing 2 mg of each into an ethanol solution (1 mL) containing 0.1% Nafion to formulate inks, which were subsequently drop-cast onto the surface of the carbon fiber papers.

In the electrocatalytic cathodic carbon dioxide reduction reaction (ECR) experiments, an Ag|AgCl electrode was utilized as the reference electrode. Linear sweep voltammetry (LSV) tests were performed at a scan rate of 5 mV s^−1^ in KHCO_3_ solution saturated with Ar or CO_2_. The frequency range for electrochemical impedance spectroscopy (EIS) measurements was from 100000 to 0.1 Hz, with the amplitude of 5 mV. Double-layer capacitance (C_dl_) was calculated through cyclic voltammetry (CV) in the non-Faradaic region to estimate the electrochemical surface area (ECSA). Before conducting each chronoamperometry (CA) test at specific potentials (including every part of the cycle test), CO_2_ was bubbled through the KHCO_3_ solution for over 30 min.

In the anodic methanol oxidation reaction (MOR) experiments, a Hg|HgO electrode was utilized as the reference electrode. LSV tests were conducted at a scan rate of 5 mV s^−1^ in KOH solution with or without 1 M methanol. The methods for EIS and the estimation of ECSA followed those of the ECR experiments. CA tests were carried out at a constant potential of 1.35 V *vs.* RHE for 20 h in KOH solution containing 1 M methanol, with the electrolyte being refreshed at the 10-hour mark.

The synchronous reactions were conducted in an H-type electrochemical cell configured in a two-electrode setup, with a proton exchange membrane separating the cathodic and anodic compartments. LSV tests were performed at a scan rate of 5 mV s^−1^ across four different paired systems: HER||OER, ECR||OER, HER||MOR, and ECR||MOR. In the ECR||MOR system aimed at the simultaneous production of formic acid, the cathode side was filled with a CO_2_-saturated 0.5 M KHCO_3_ solution, while the anode side contained a 1 M KOH solution with 1 M methanol.

**Product analysis**

The liquid product (formic acid) from the cathodic and anodic reactions was analyzed using high-performance liquid chromatography (HPLC, Shimadzu) equipped with a Sepax Carbomix H-NP column and a UV detector at 220 nm. The mobile phase consisted of a 2.5 mM H_2_SO_4_ solution, with a flow rate of 0.6 mL min^−1^, and the column temperature was maintained at 55 ℃. The gas products from the cathodic reaction were quantified using gas chromatography (Techcomp GC 7900) equipped with a TDX-01 column. Ar was used as the carrier gas, with a thermal conductivity detector (TCD) for H_2_ detection, and a flame ionization detector (FID) for CO and CH_4_ detection.

**Nernst equation**

*E*_RHE_ = *E*_Ag|AgCl_ + 0.059 × pH + 0.197 V

*E*_RHE_ = *E*_Hg|HgO_ + 0.059 × pH + 0.098 V

In the equations, *E*_RHE_ represents the potential of reversible hydrogen electrode (RHE), pH is from KHCO_3_ solution saturated with Ar or CO_2_, as well as from KOH solution with and without methanol, and *E*_Ag|AgCl_ (*E*_Hg|HgO_) is the measurement of potential relative to reference electrode tested by the electrochemical workstation.

**Faradaic efficiency**

Faradaic efficiency = $\frac{Q_{\mathrm{product}}}{Q}$ × 100% = $\frac{mnF}{Q}$ × 100%

In the equation, *m* represents the actual mole number of the product, *n* denotes the corresponding number of electrons involved in the cathodic reaction (2 for HCOOH, H_2_ and CO and 8 for CH_4_) and anodic reaction (2 for HCOOH), F signifies the Faraday constant (96485 C mol^−1^), and *Q* corresponds to the total electric charge passed through the circuit.

**Cathodic energy efficiency**

Cathodic energy efficiency = $\frac{1.23 V-E_{f}}{1.23 V-E}$ × Faradaic efficiency

In the equation, *E*_f_ denotes the thermodynamic potential for the conversion of CO_2_ to formic acid (−0.2 V *vs.* RHE), while *E* (V *vs.* RHE) represents the applied potential during the reaction.

**Theoretical Calculations**

The calculations of the change in Gibbs free energy for carbon dioxide reduction on Cu (111) and CuBi (012) were implemented using the Cambridge sequential total energy package (CASTEP) module of Materials Studio software.^[1-3]^ Exchange-correlation functions are described by generalized gradient approximation (GGA) with Perdew–Burke–Ernzerhof (PBE) pseudopotentials, and the cutoff energy was set to 400 eV. Geometric optimization of all the atomic models was carried out before the energy calculations, and the following convergent tolerances were set: the changes of energy, the maximum stress and the maximum displacement were 2 × 10^−5^ eV atom^−1^, 0.1 GPa and 0.002 Å, respectively. To avoid artificial interactions caused by periodicity between adjacent layers in adsorption models, the computational models are set with 10 Å vacuum layers. The calculation details of the reaction free energy (ΔG) for each step are as follows ^[4,5]:^

$\text{∆G=∆E-T∆S+∆ZPE}$ (1)

$\text{∆E=}\text{E}_{\text{2}}\text{-}\text{E}_{\text{1}}$ (2)

where E_1_, E_2_, △S, T, and △ZPE are the energies of the corresponding total energy of the system before the reaction step, the total energy of the system after the reaction step, the corrective changes in entropy, Kelvin temperature and the corrective changes in zero-point energies, respectively.

**Supplementary Figures**


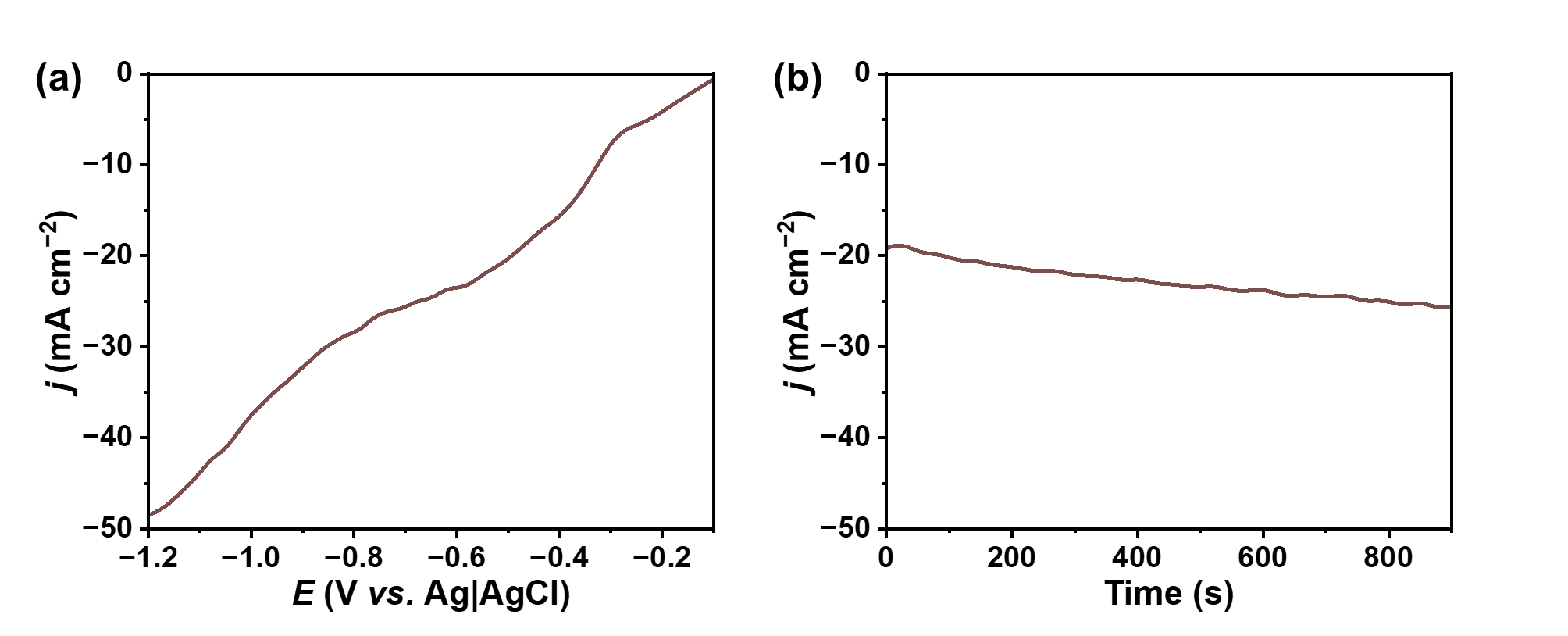


**Figure S1.** (a) LSV curve of the carbon fiber paper electrode in the electrodeposition. (b) Current-time curve during the electrodeposition synthesis process.


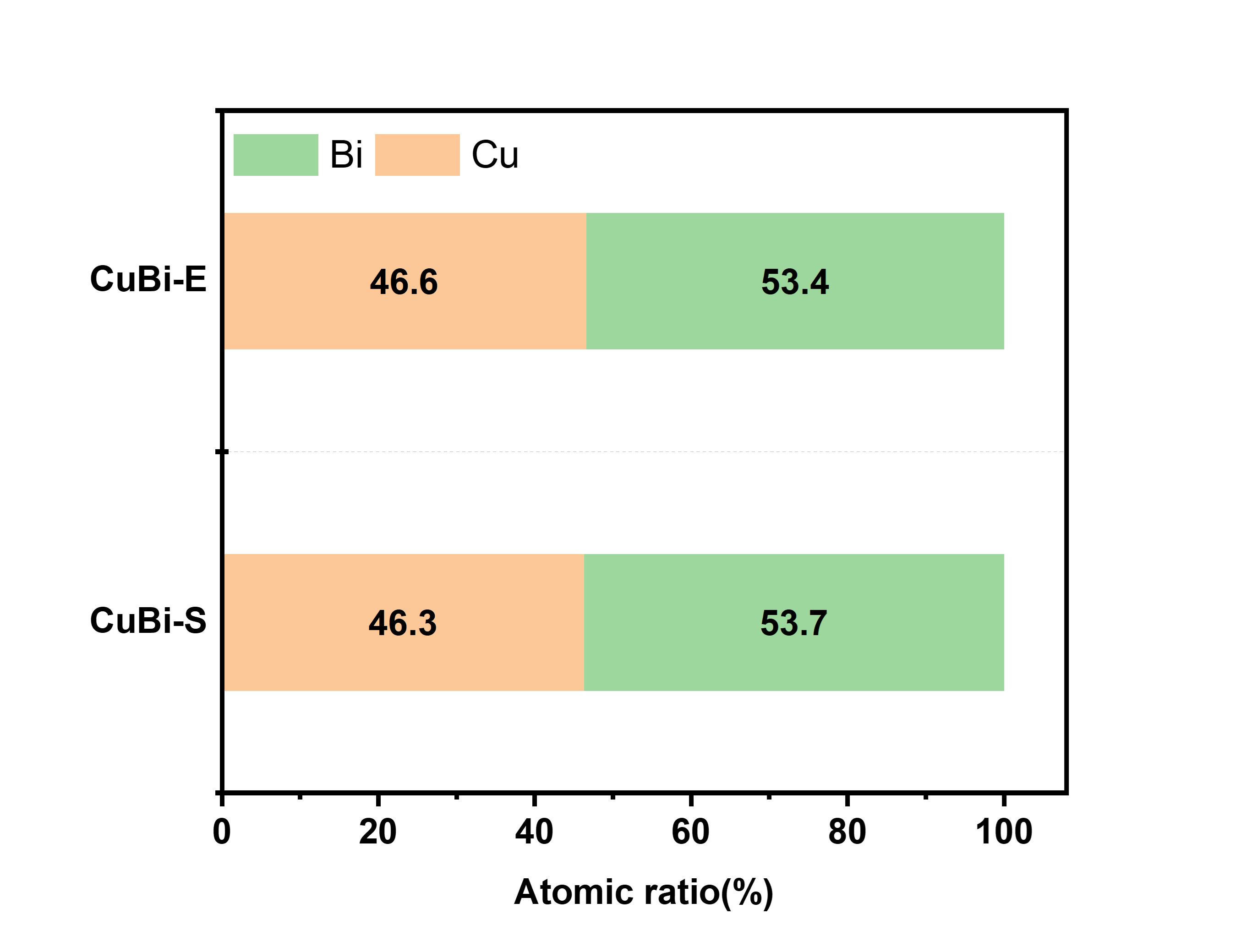


**Figure S2.** The atomic ratio of (a) CuBi-E, (b) CuBi-S measured by ICP-OES.


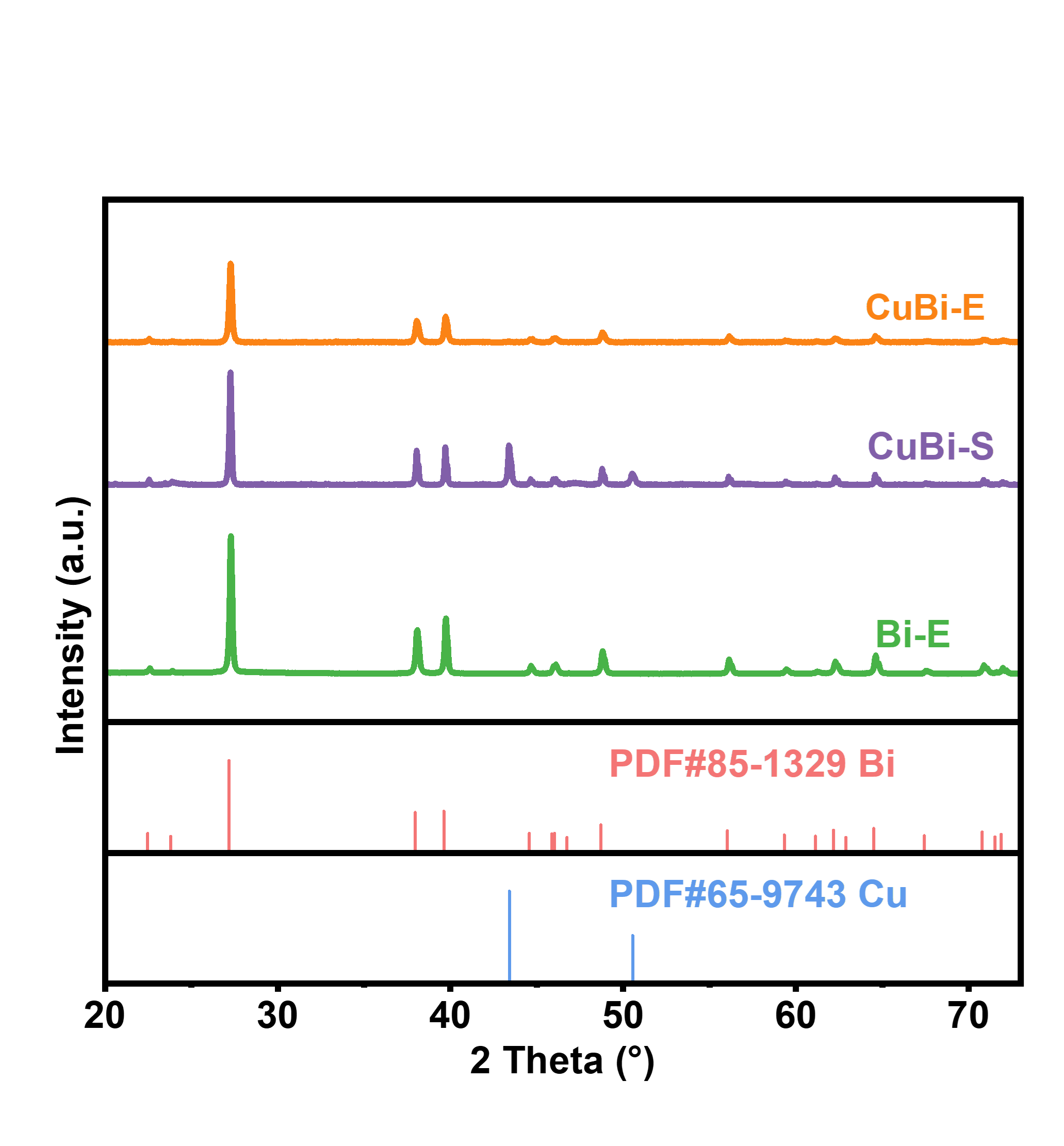


**Figure S3.** XRD patterns of CuBi-E, CuBi-S and Bi-E catalysts.


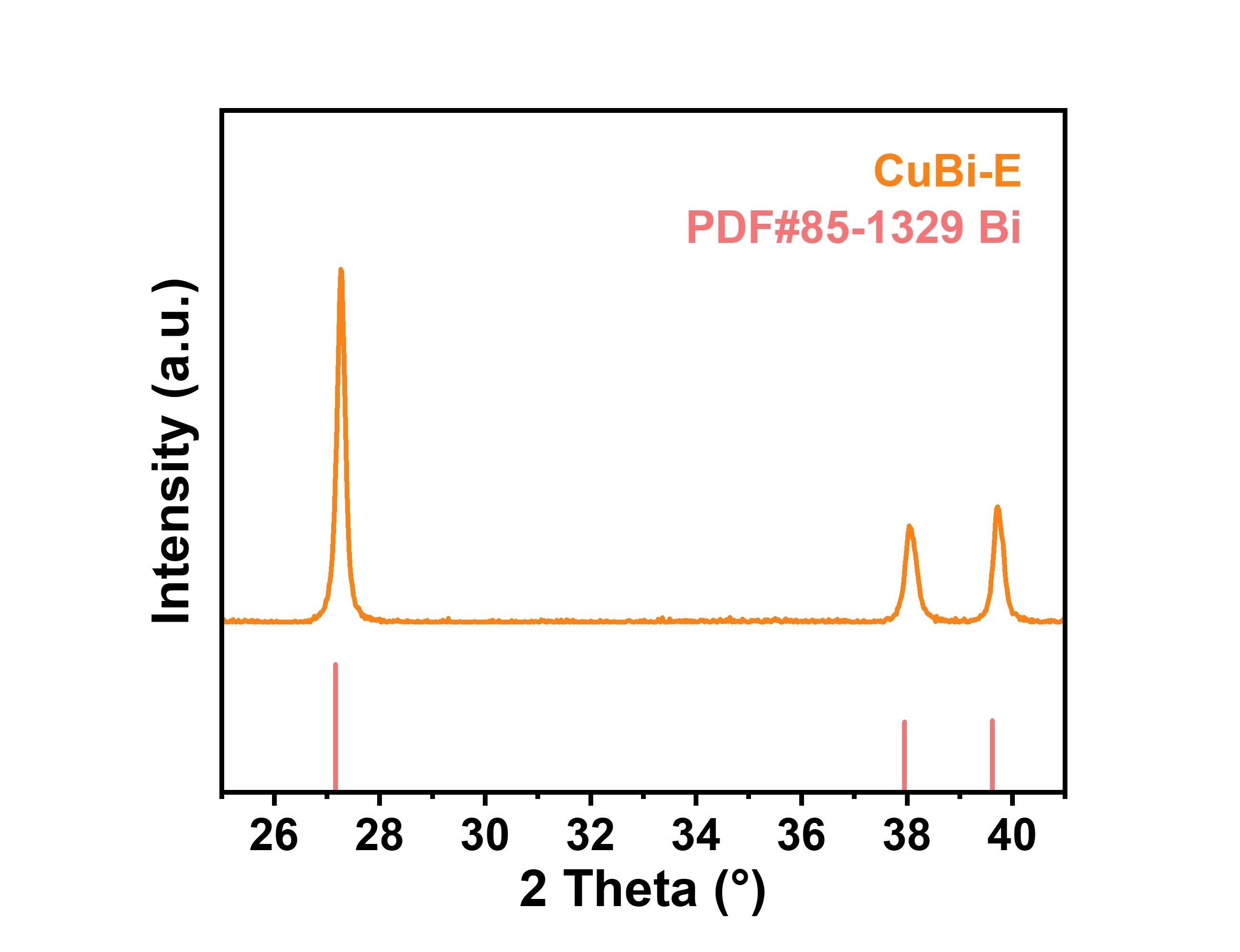


**Figure S4.** The enlarged XRD patterns of CuBi-E.


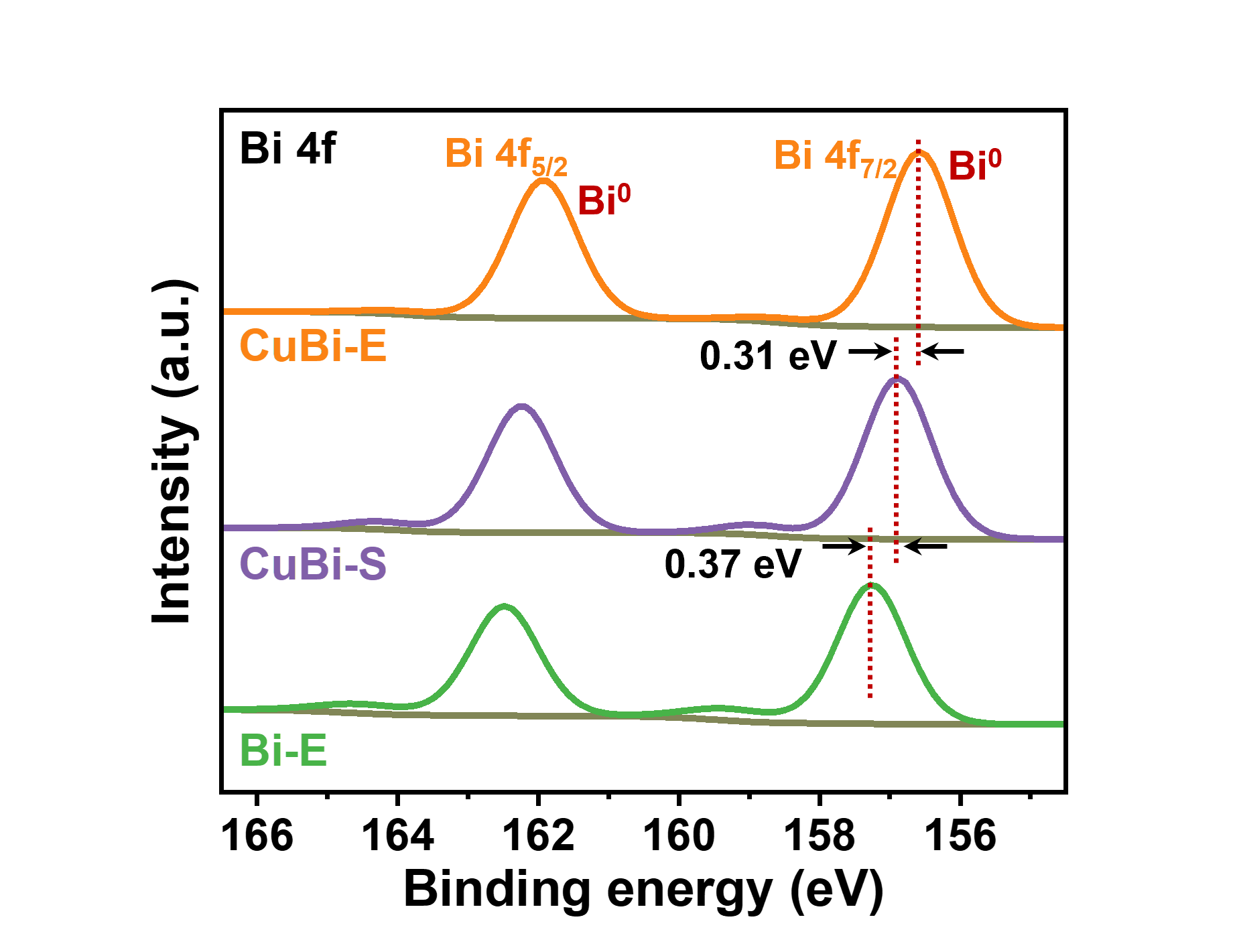


**Figure S5.** Bi 4f XPS spectra of CuBi-E, CuBi-S and Bi-E catalysts.


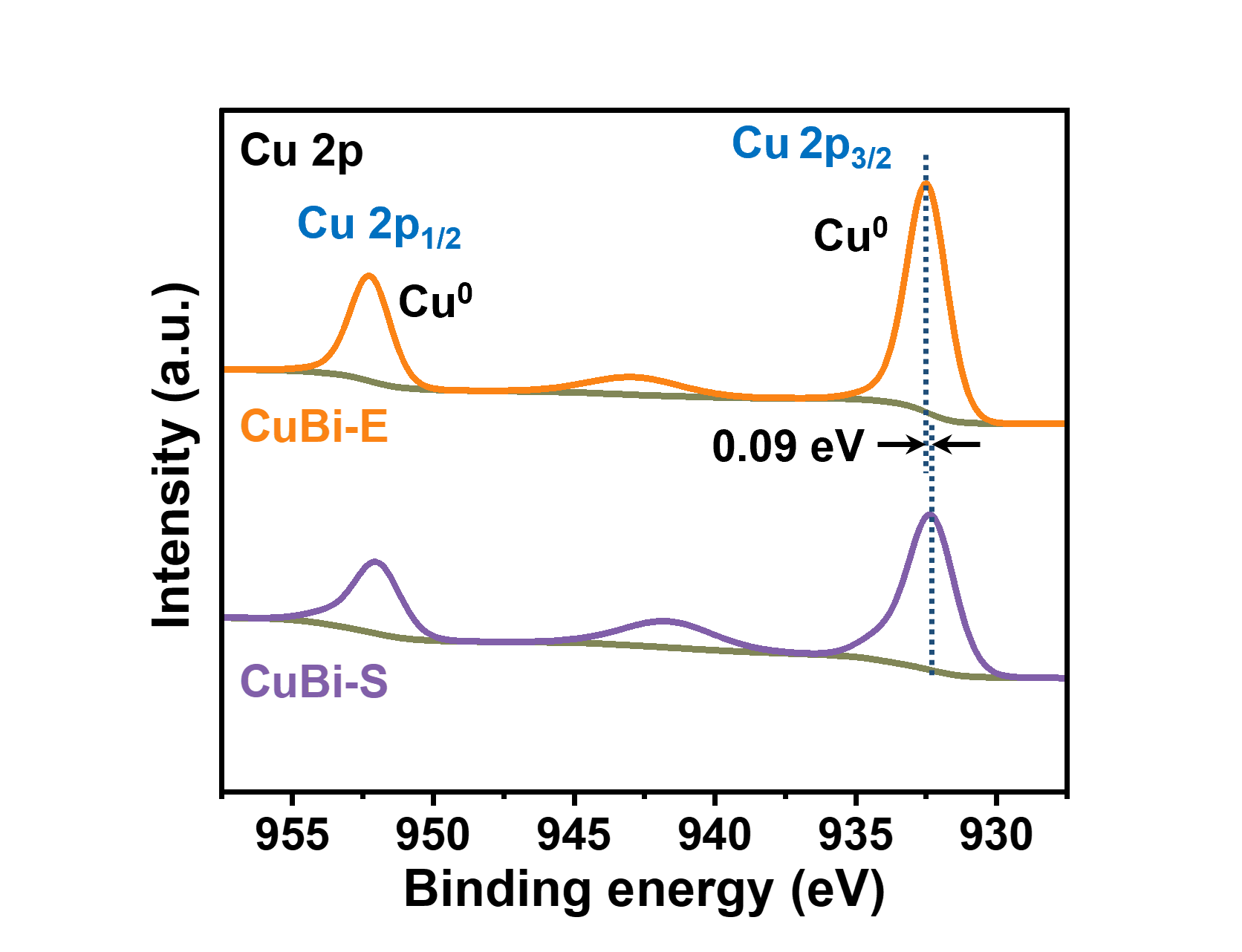


**Figure S6.** Cu 2p XPS spectra of CuBi-E and CuBi-S catalysts.


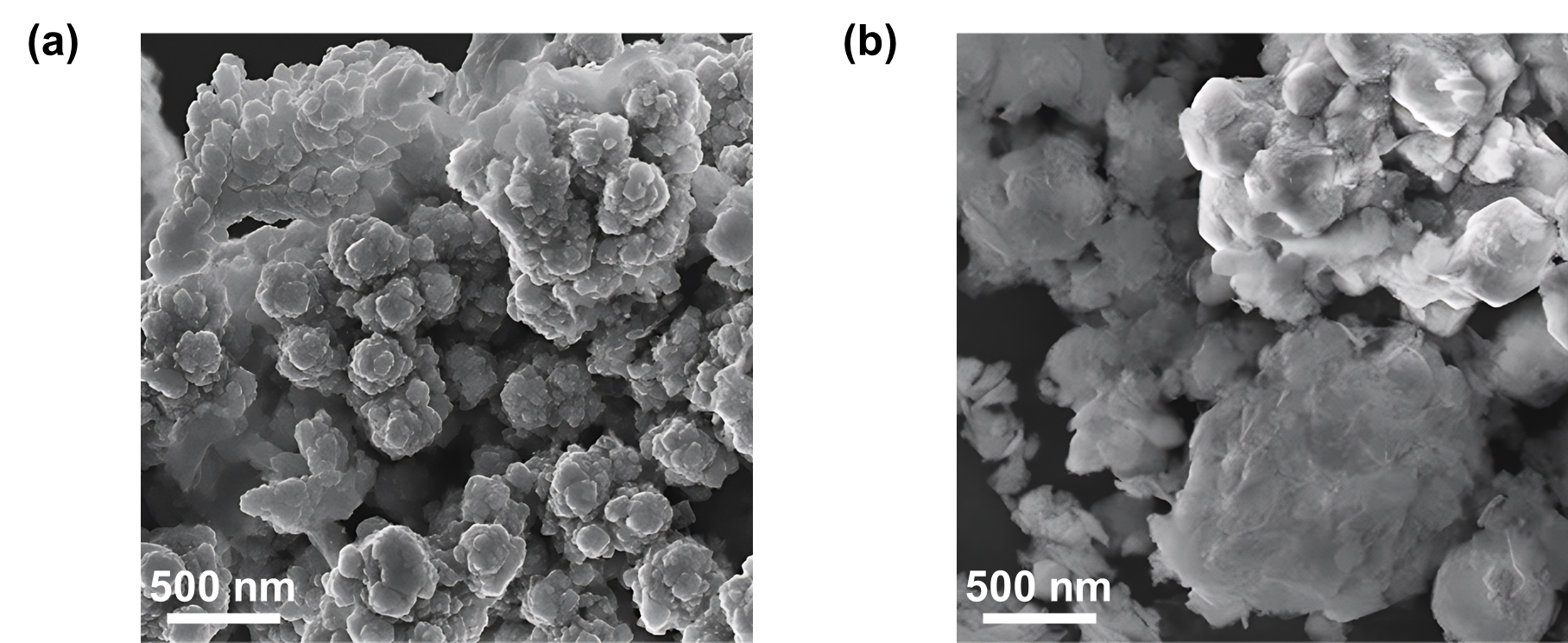


**Figure S7.** SEM images of (a) CuBi-E and (b) CuBi-S.


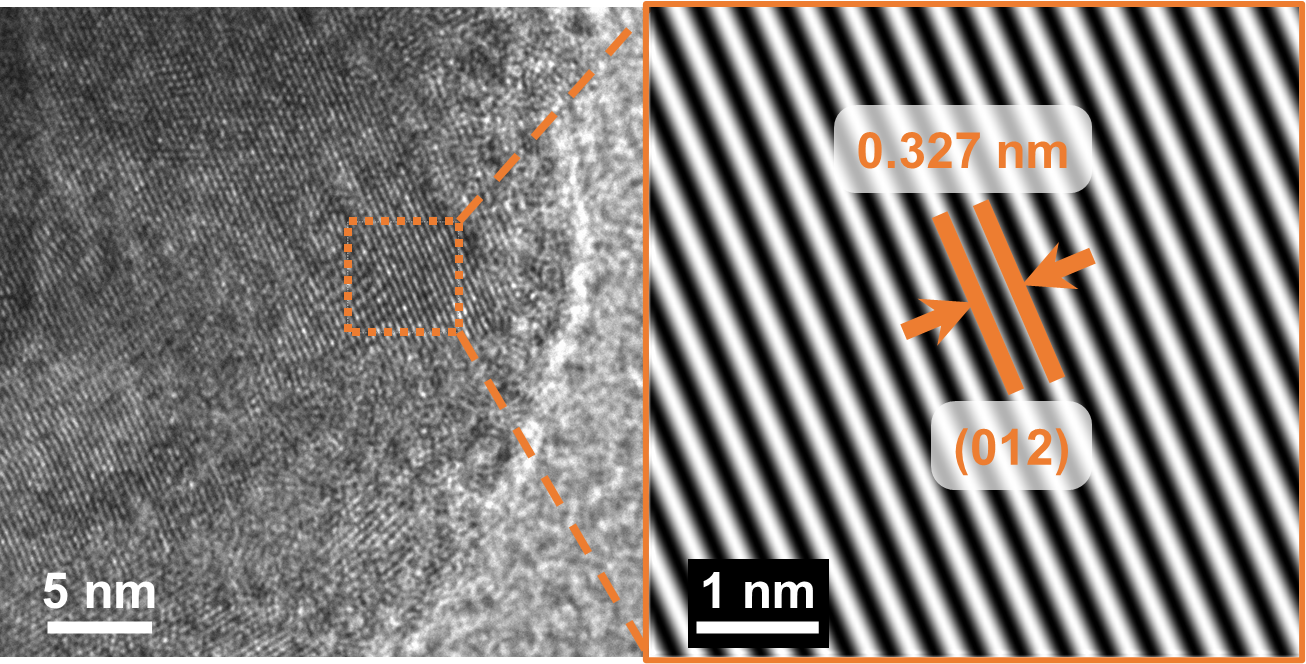


**Figure S8.** HR-TEM and corresponding magnified inverse FFT images of CuBi-E.


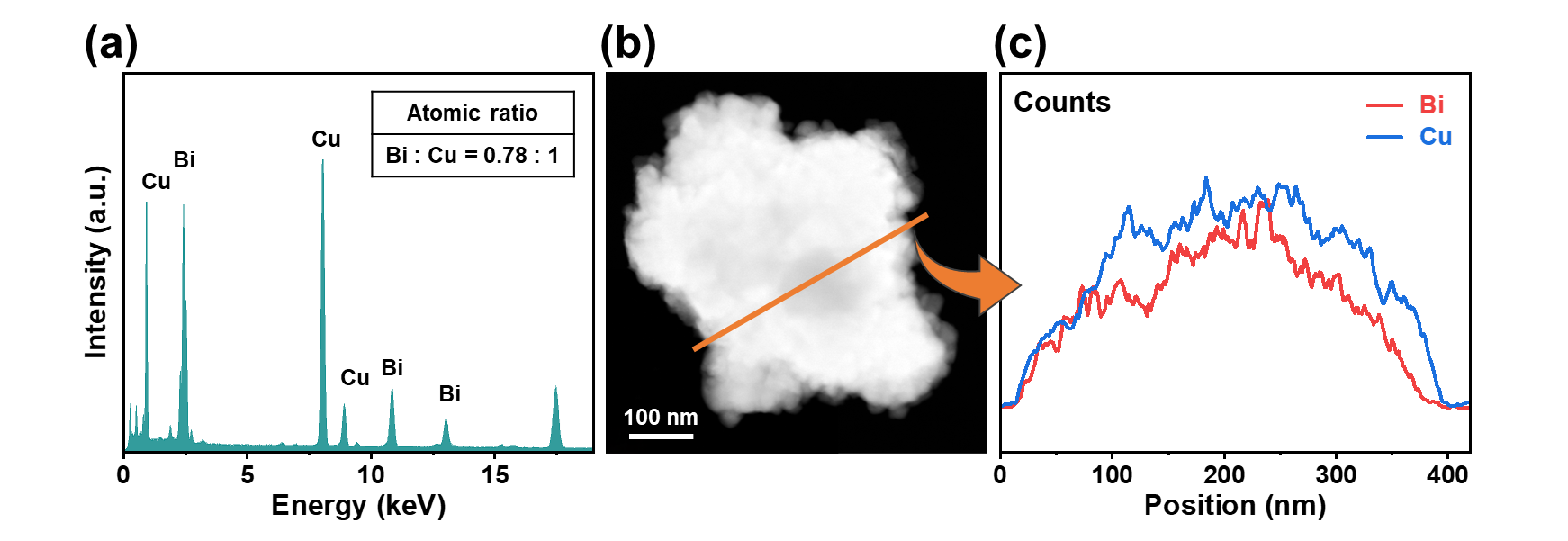


**Figure S9.** (a) EDS spectrum of CuBi-E. (b) STEM image and (c) corresponding line scanning profiles.


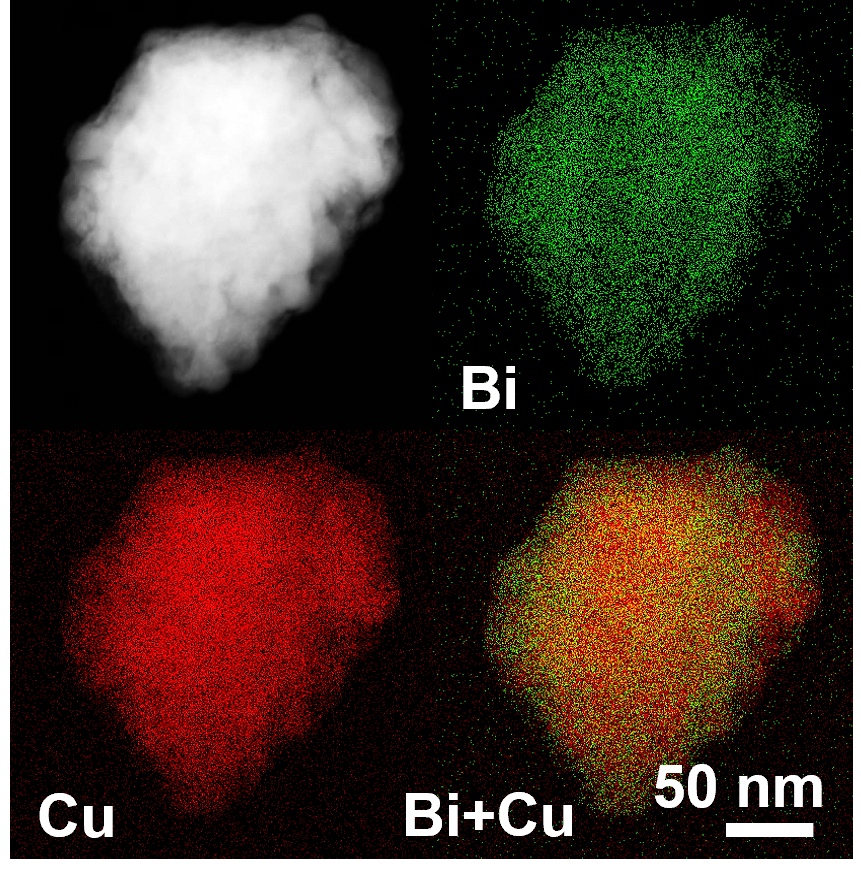


**Figure S10.** STEM-mapping images of CuBi-E catalyst.


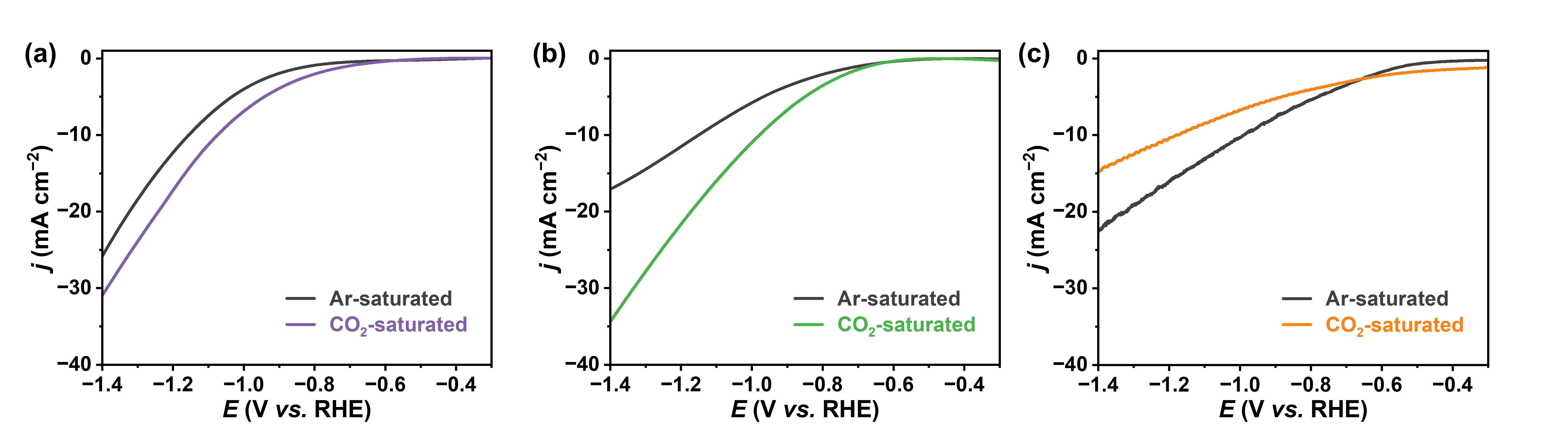


**Figure S11.** LSV curves of (a) CuBi-S (b) Bi-E and (c) Cu-E in 0.1 M KHCO_3_ solution saturated with Ar or CO_2_.


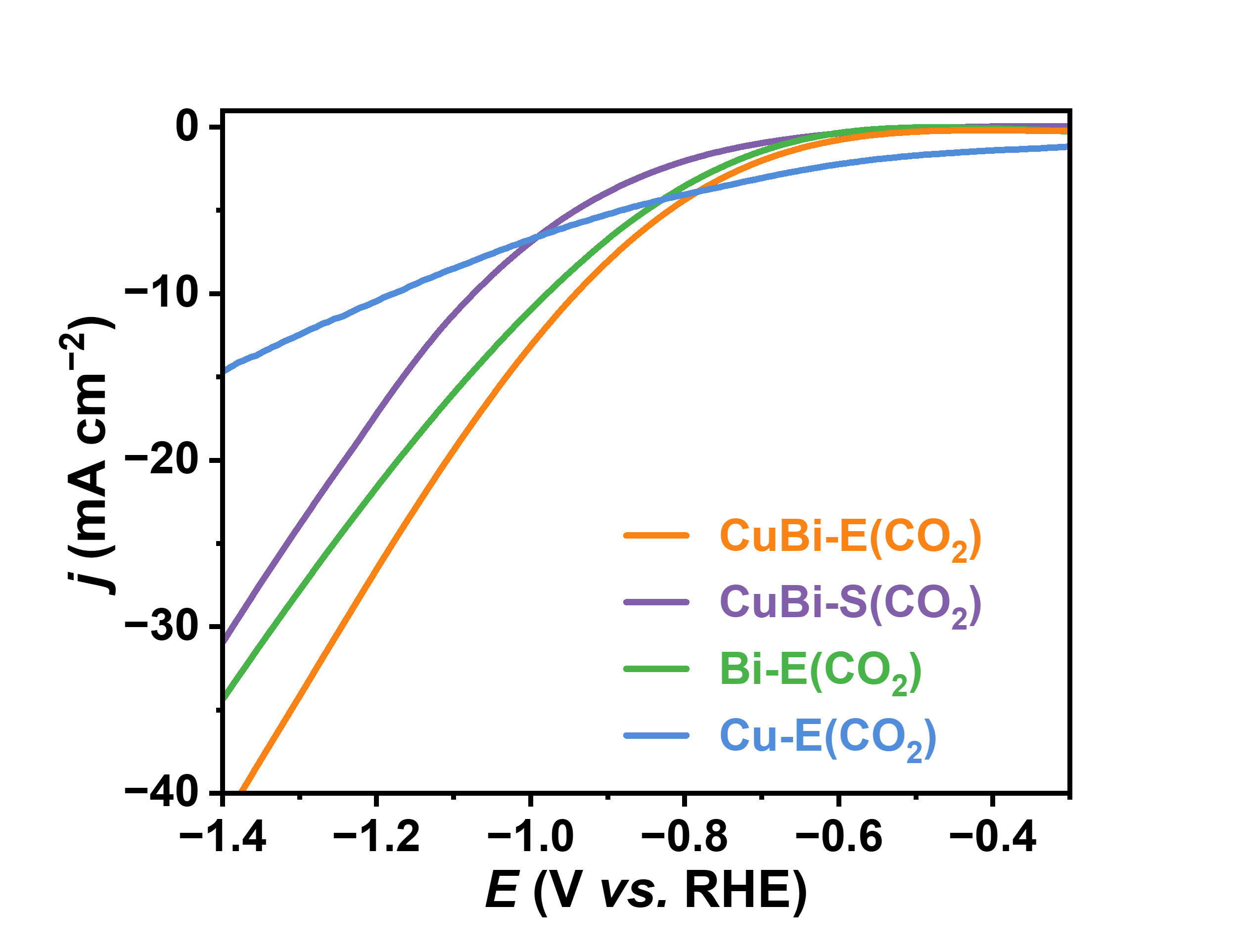


**Figure S12.** LSV curves of CuBi-E, CuBi-S, Bi-E and Bi-E in CO_2_-saturated 0.1 M KHCO_3_ solution at the united scale.


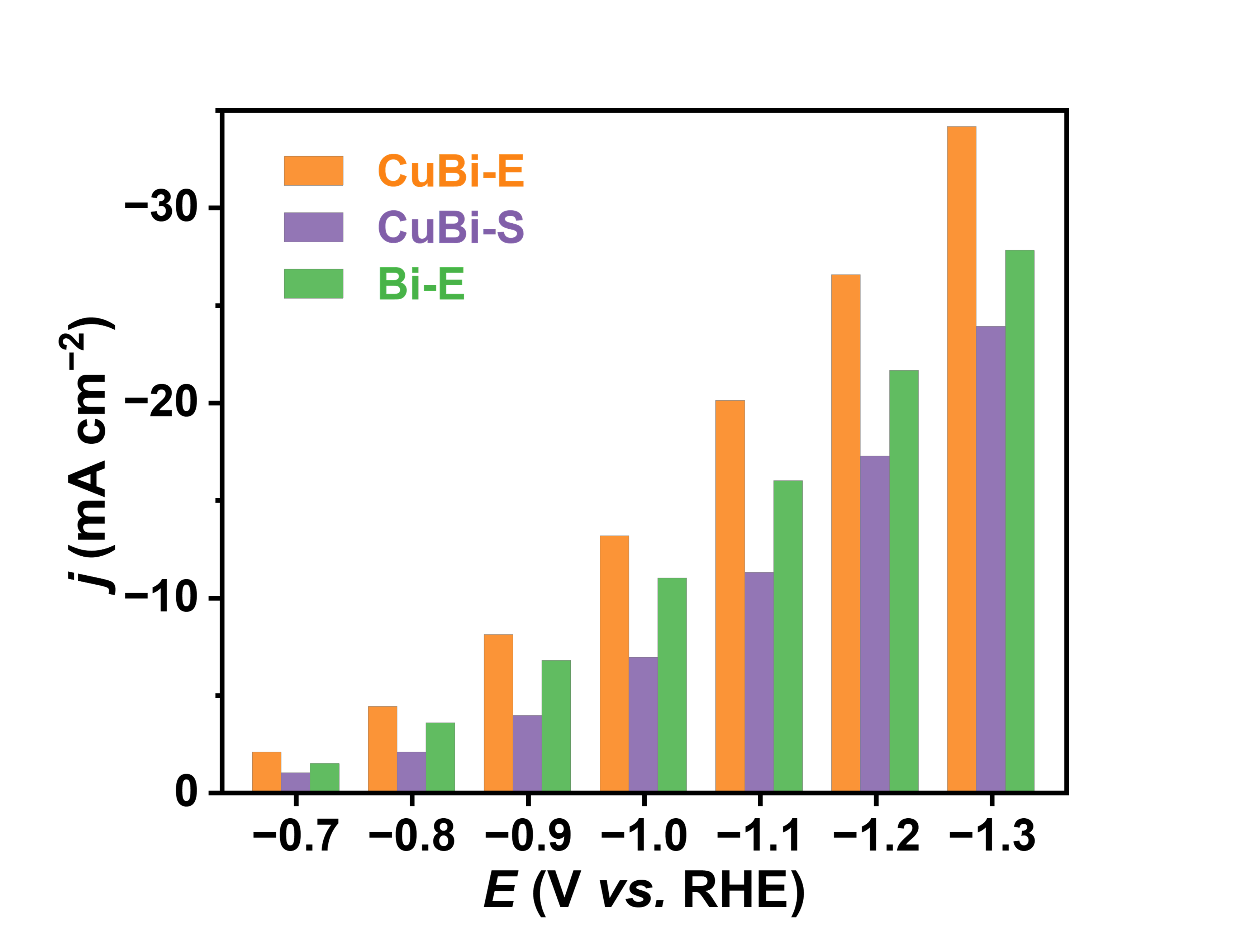


**Figure S13.** The relationship between the current density and potential of CuBi-E, CuBi-S and Bi-E was obtained from LSV curves in CO_2_-saturated 0.1 M KHCO_3_ solution.


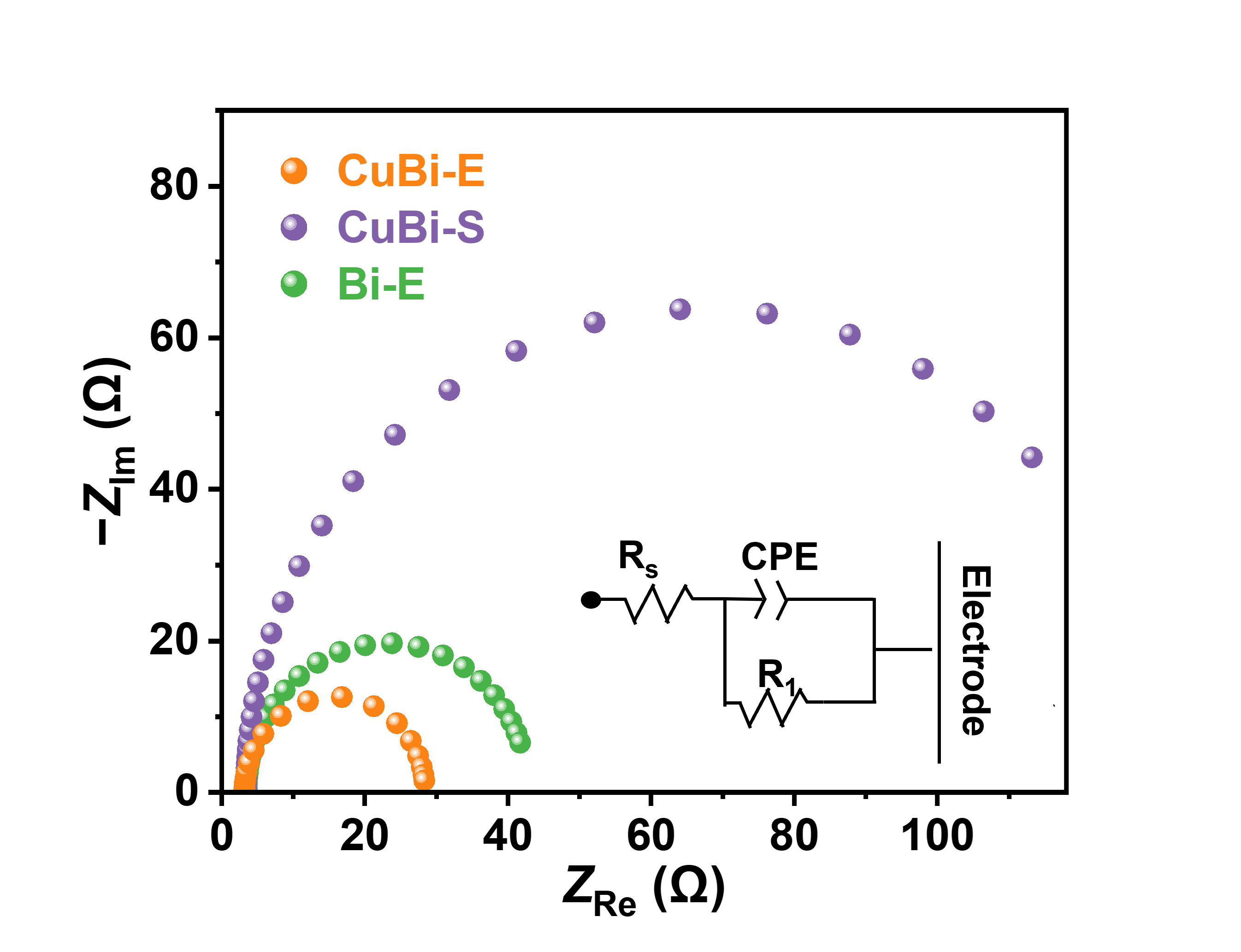


**Figure S14.** Nyquist plots of CuBi-E, CuBi-S and Bi-E in the ECR test system (The illustration shows the equivalent circuit diagram during fitting).


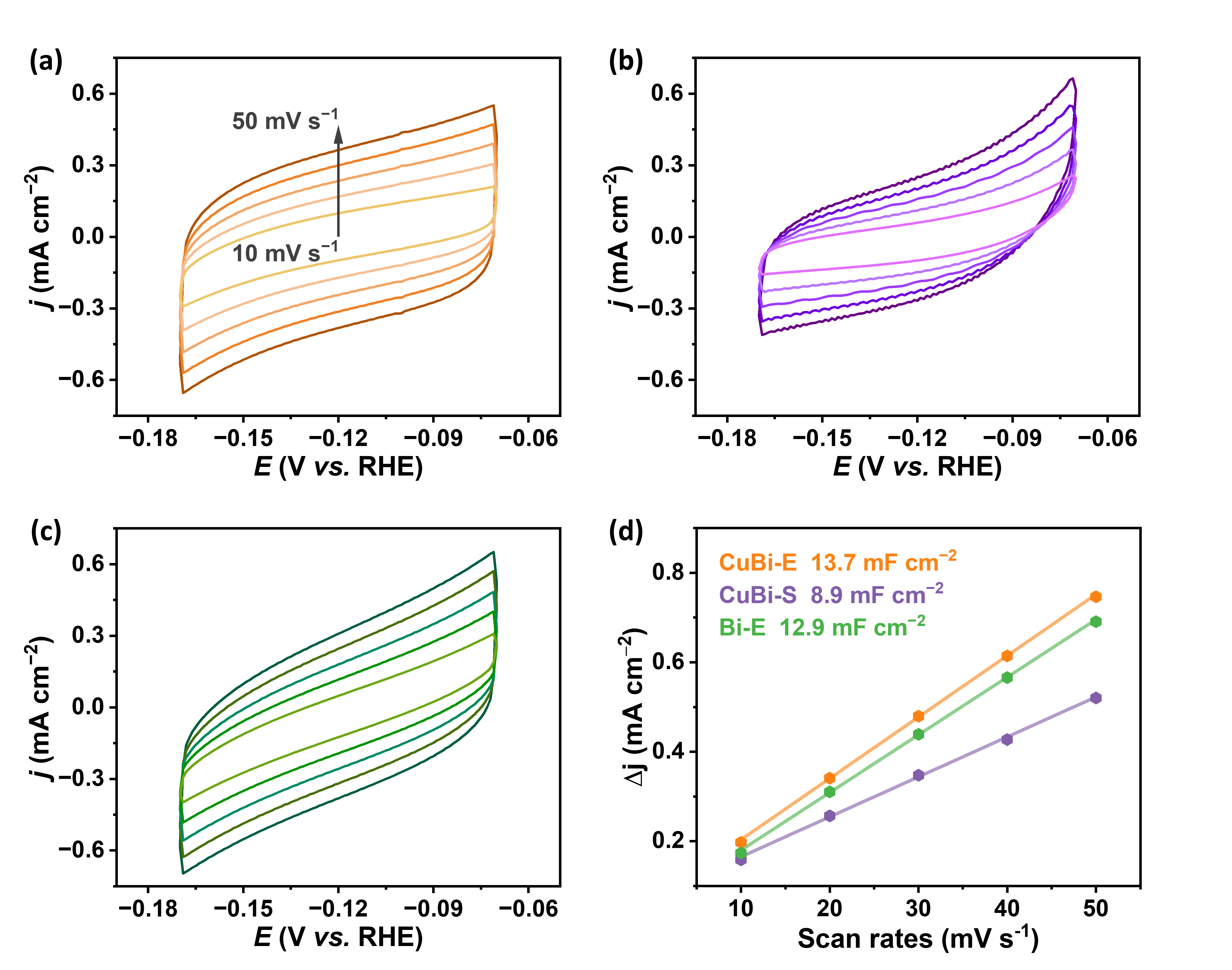


**Figure S15.** CV curves of (a) CuBi-E, (b) CuBi-S and (c) Bi-E at different scan rates and (d) calculated double-layer capacitance.

**Figure S16**. LSV curves of CuBi-E, CuBi-S, Bi-E and Bi-E in CO_2_-saturated 0.1 M KHCO_3_ solution normalized by ECSA.


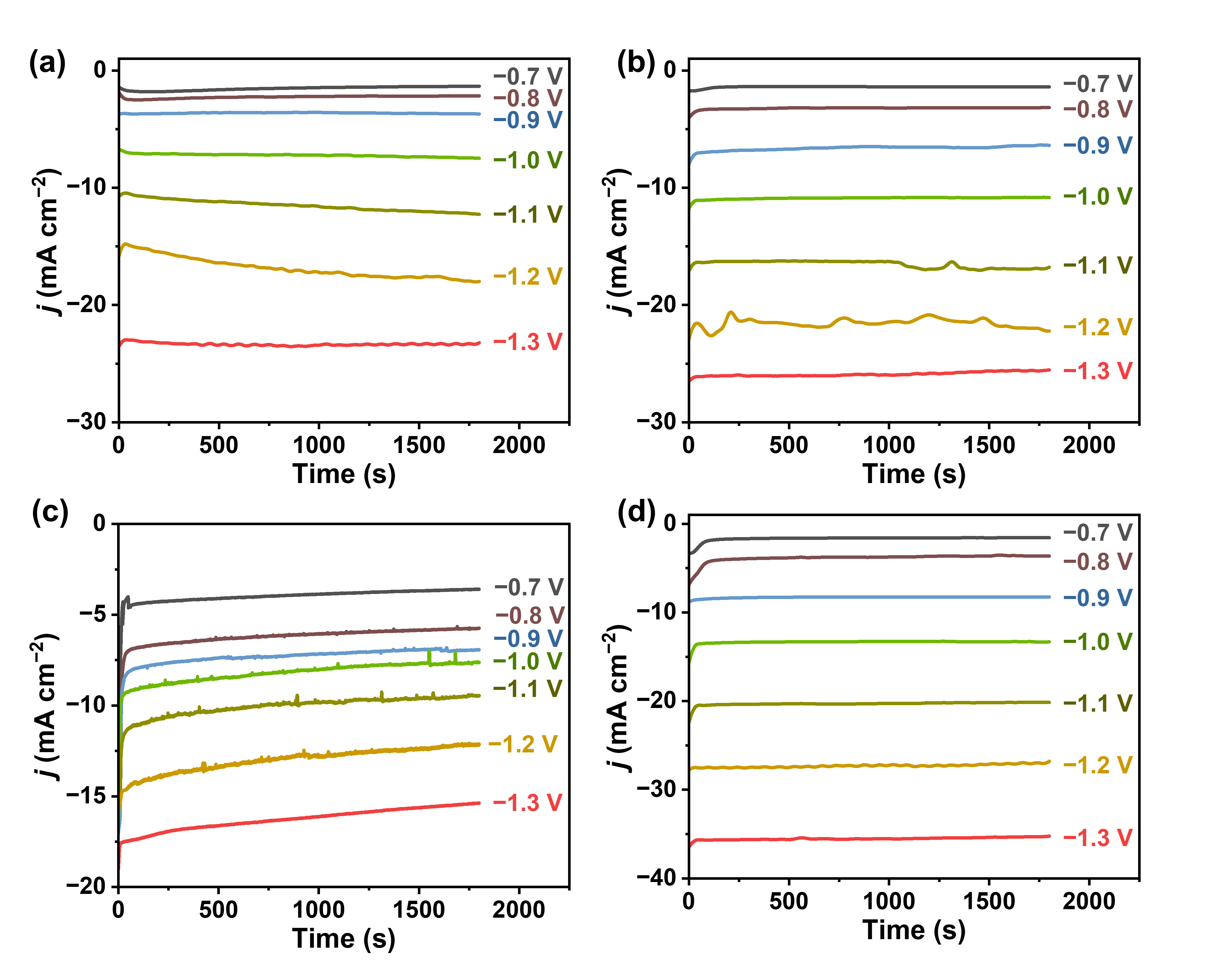


**Figure S17.** CA test curves of (a) CuBi-S, (b) Bi-E, (c) Cu-E and (d) CuBi-E at different potentials.


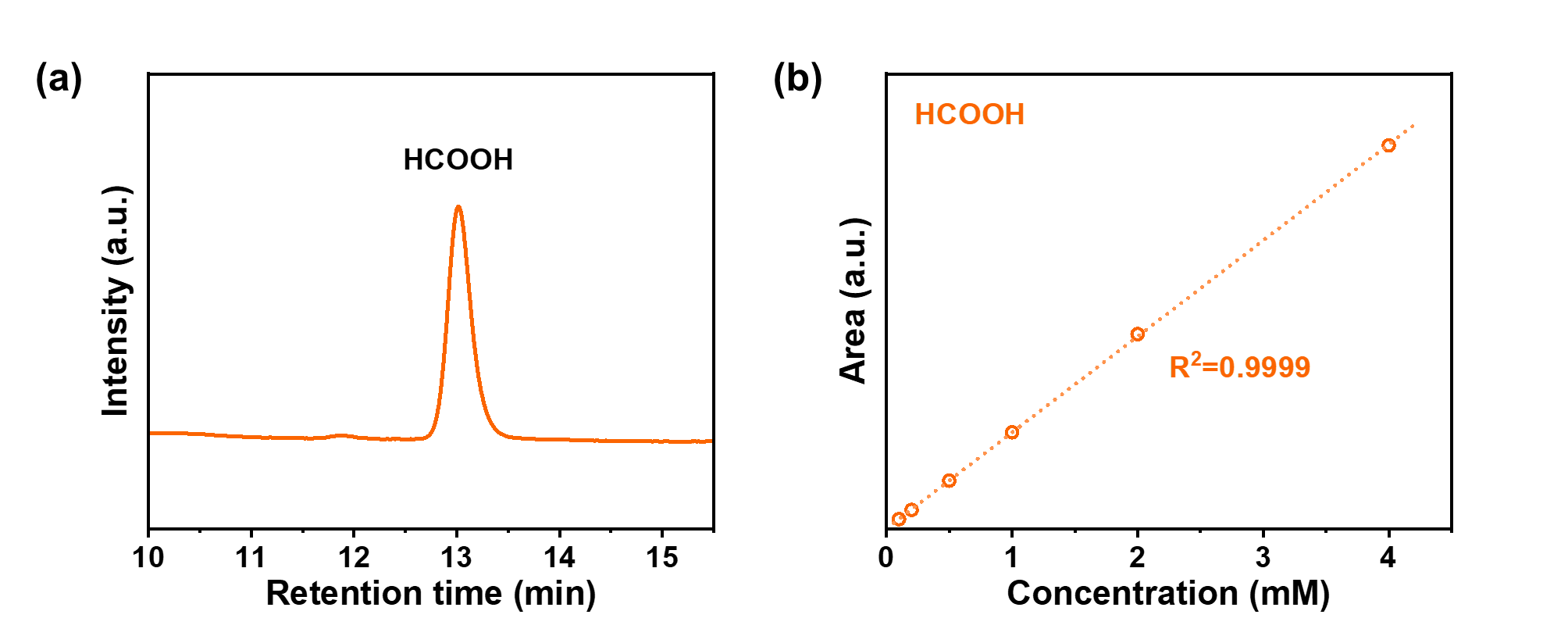


**Figure S18.** (a) Schematic diagram of the peak position of formic acid in the liquid chromatogram. (b) Standard quantity relationship curve between the intensity and the concentration of formic acid.


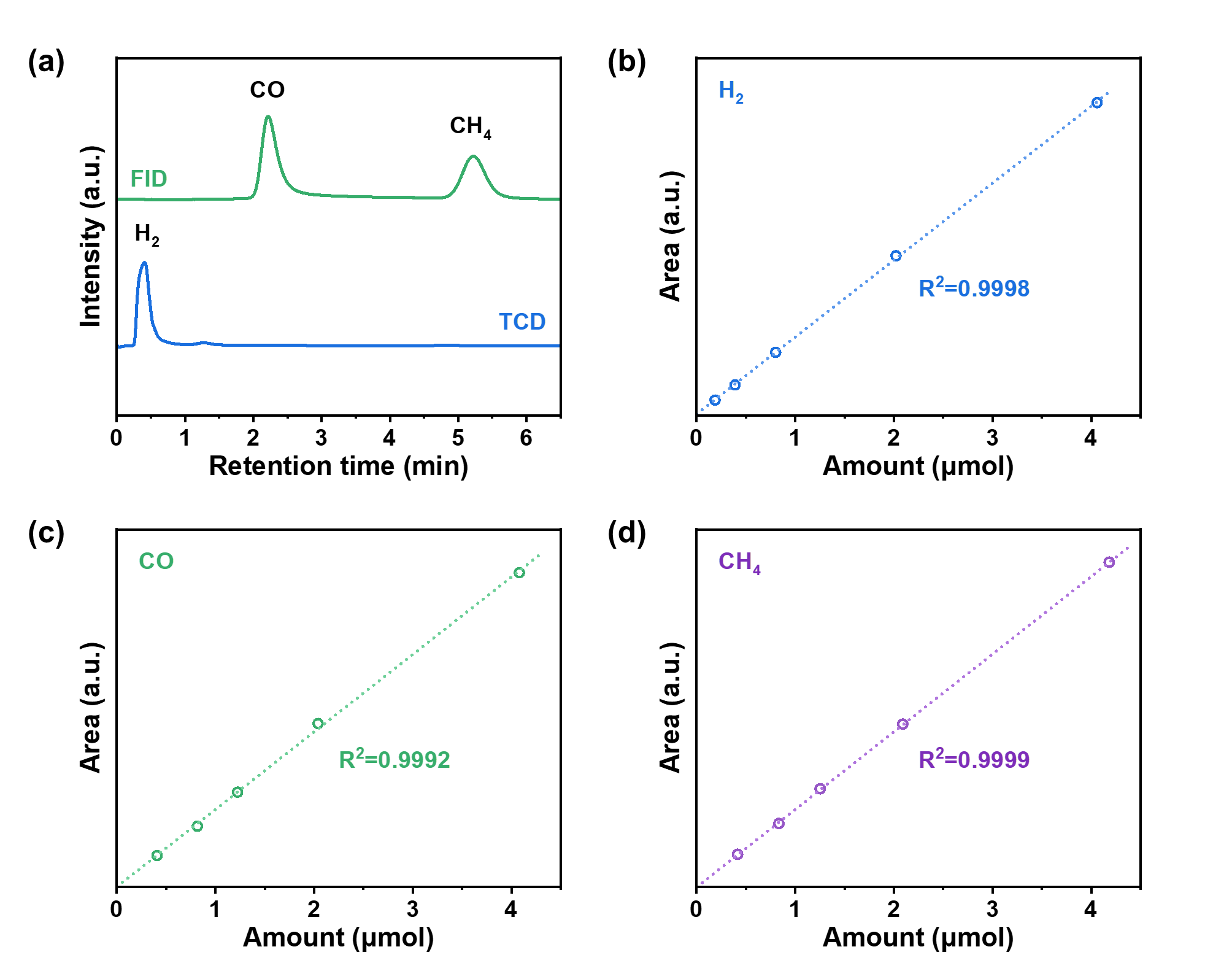


**Figure S19.** (a) Schematic diagram of the peak positions of H_2_, CO and CH_4_ in the gas chromatogram. Standard quantity relationship curve between the intensity and the amount of substance of (b) H_2_, (c) CO and (d) CH_4_.


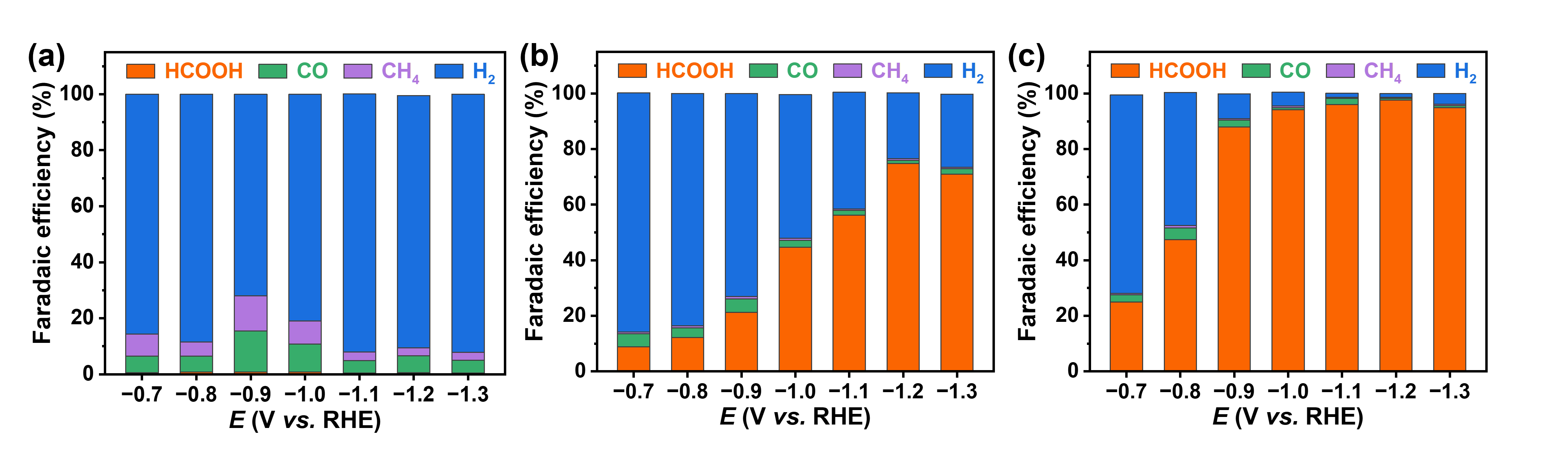


**Figure S20.** Faradaic efficiency of products at different potentials for (a) Cu-E, (b) CuBi-S and (b) Bi-E.


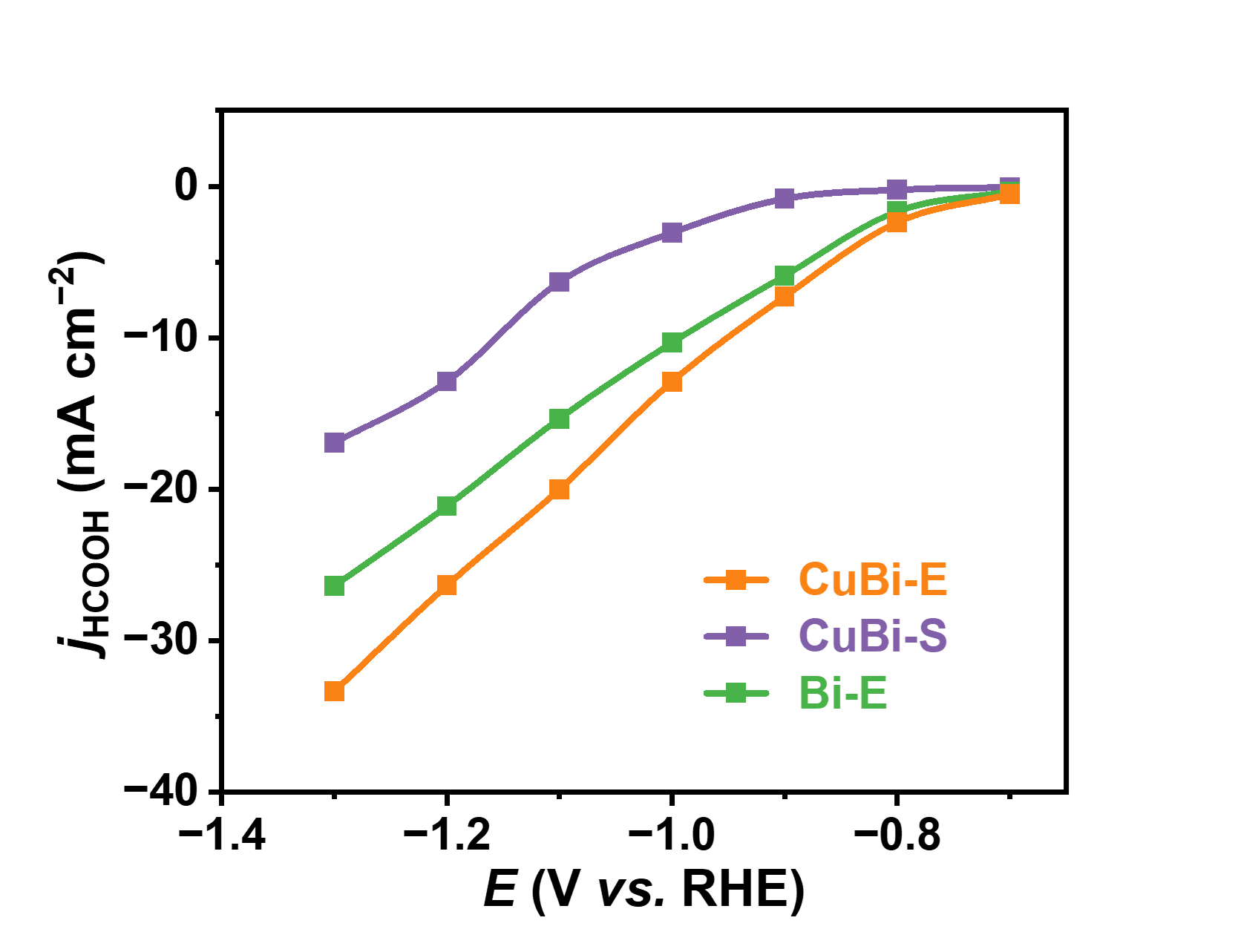


**Figure S21.** Formic acid partial current density against applied potential over CuBi-E, CuBi-S and Bi-E.


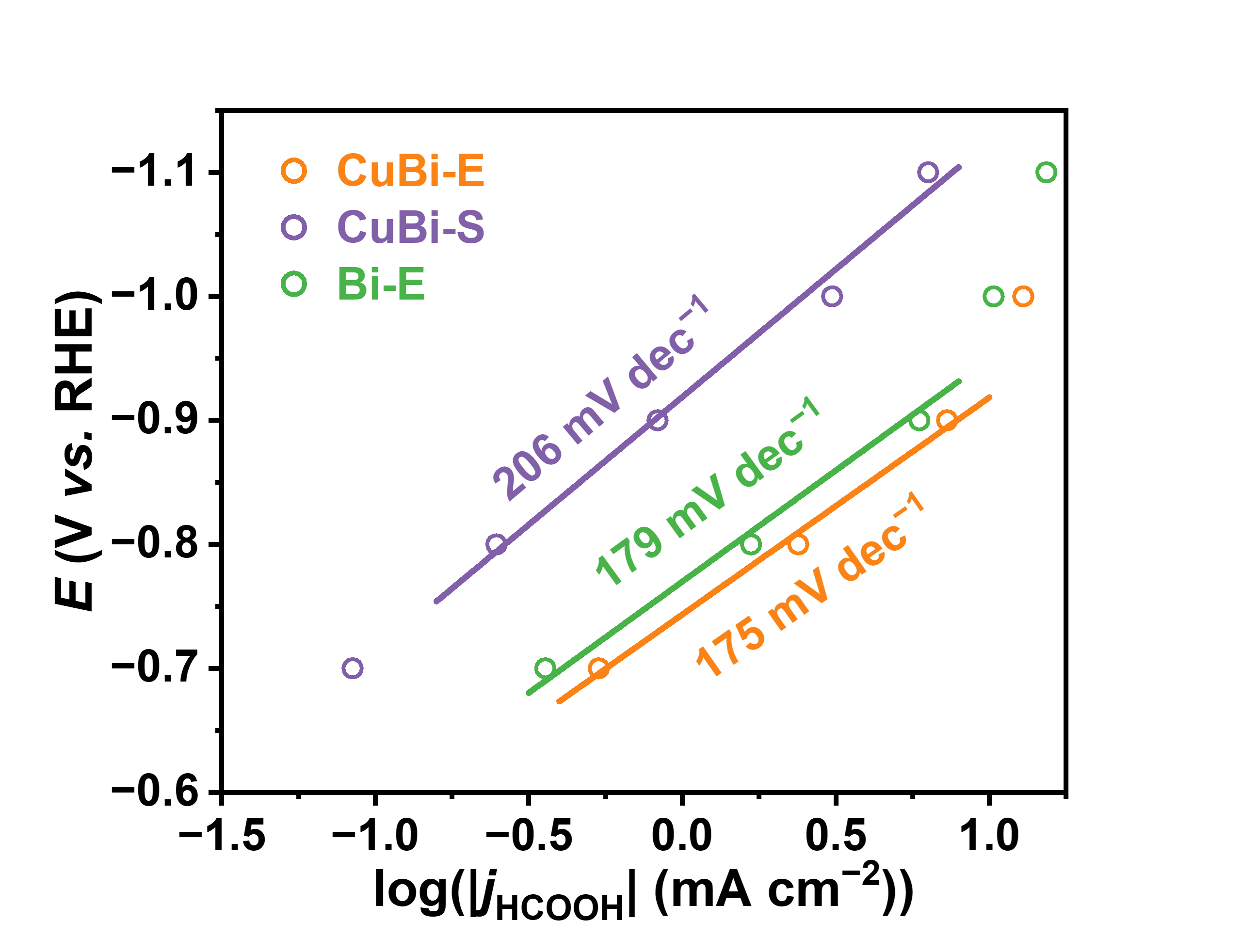


**Figure S22.** Tafel slopes of CuBi-E, CuBi-S and Bi-E in the ECR system.


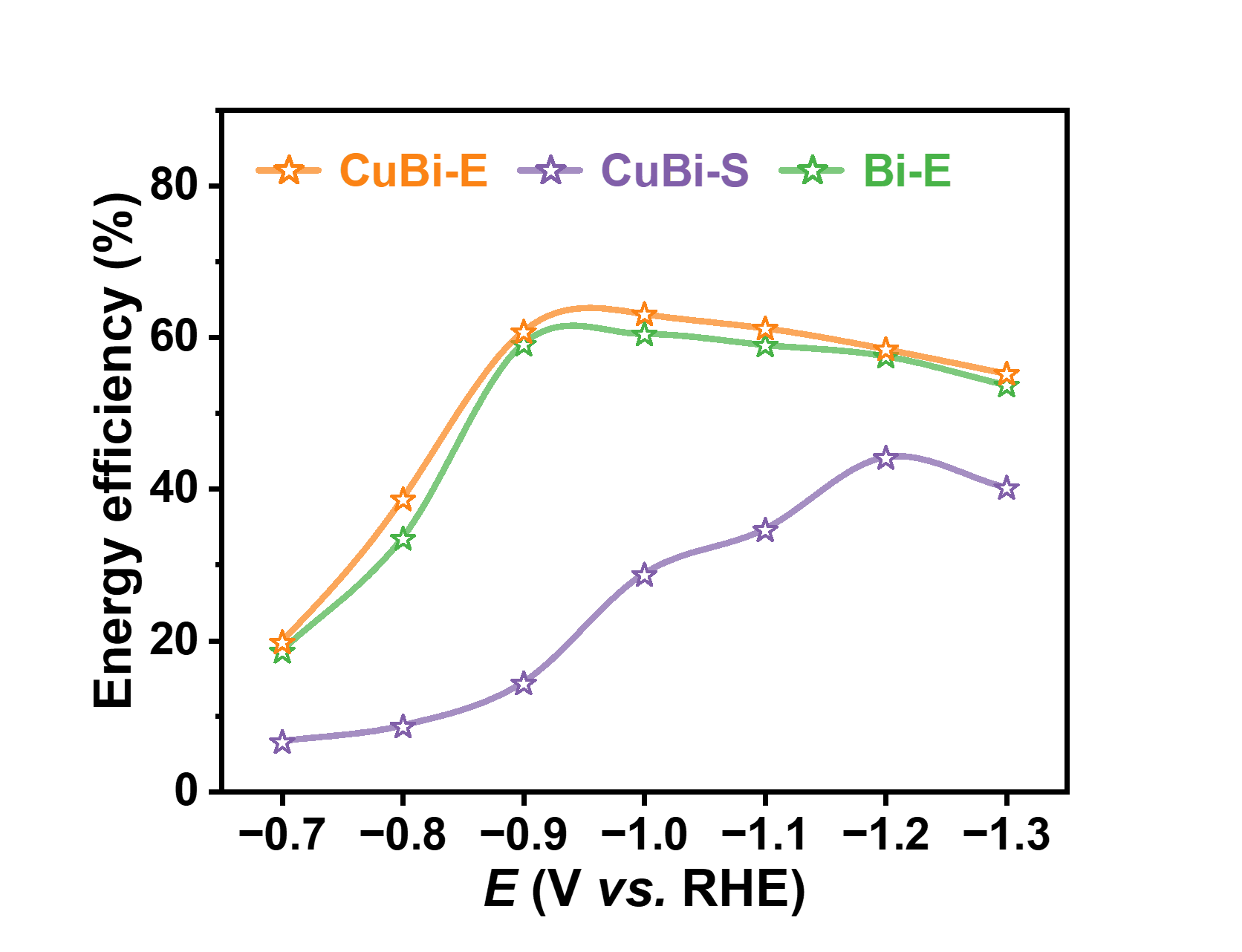


**Figure S23.** The energy efficiency of CO_2_ conversion to formic acid on CuBi-E, CuBi-S and Bi-E.


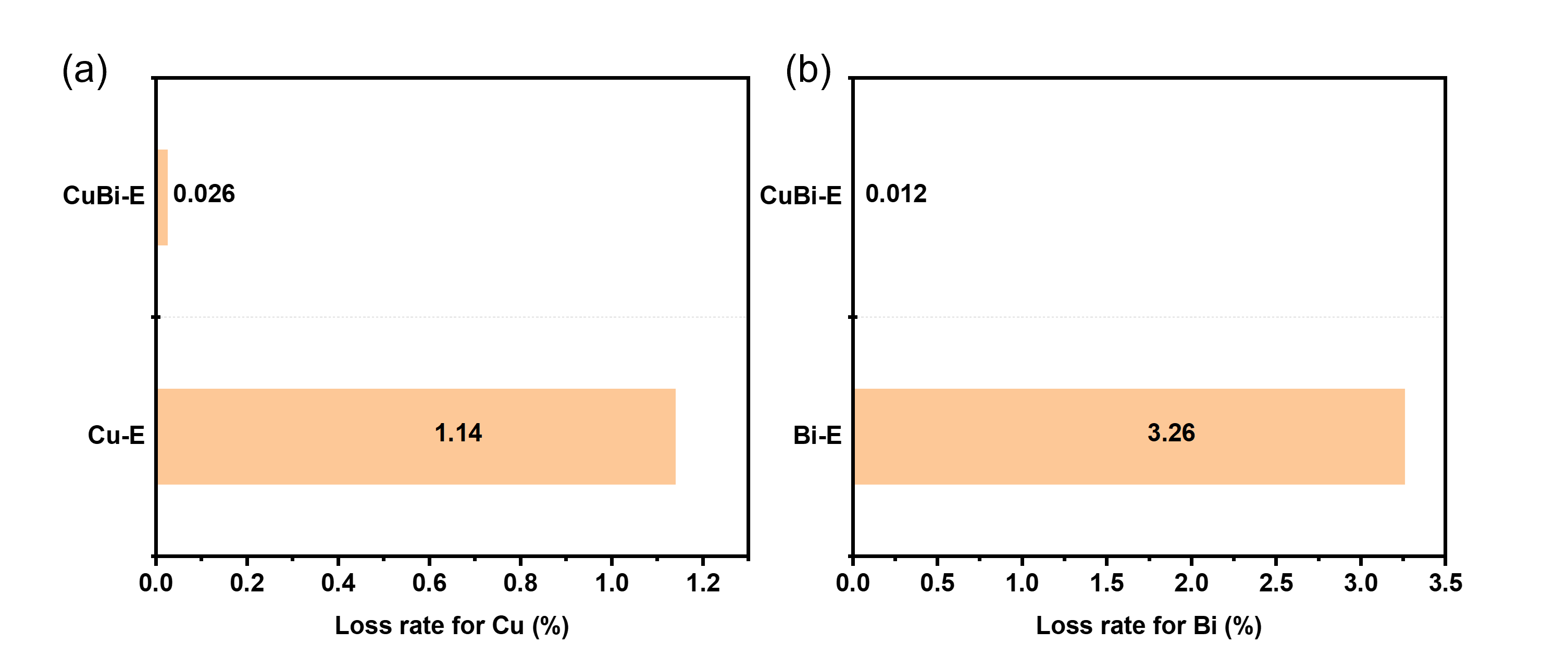


**Figure S24.** The loss rate of Cu and Bi from Cu-E, Bi-E and CuBi-E catalysts tested by ICP-OES after 6 h ECR.

**Figure S25.** LSV curves of CuBi-E in CO_2_-saturated 0.1 M KHCO_3_ solution during before and after cyclic experiment.


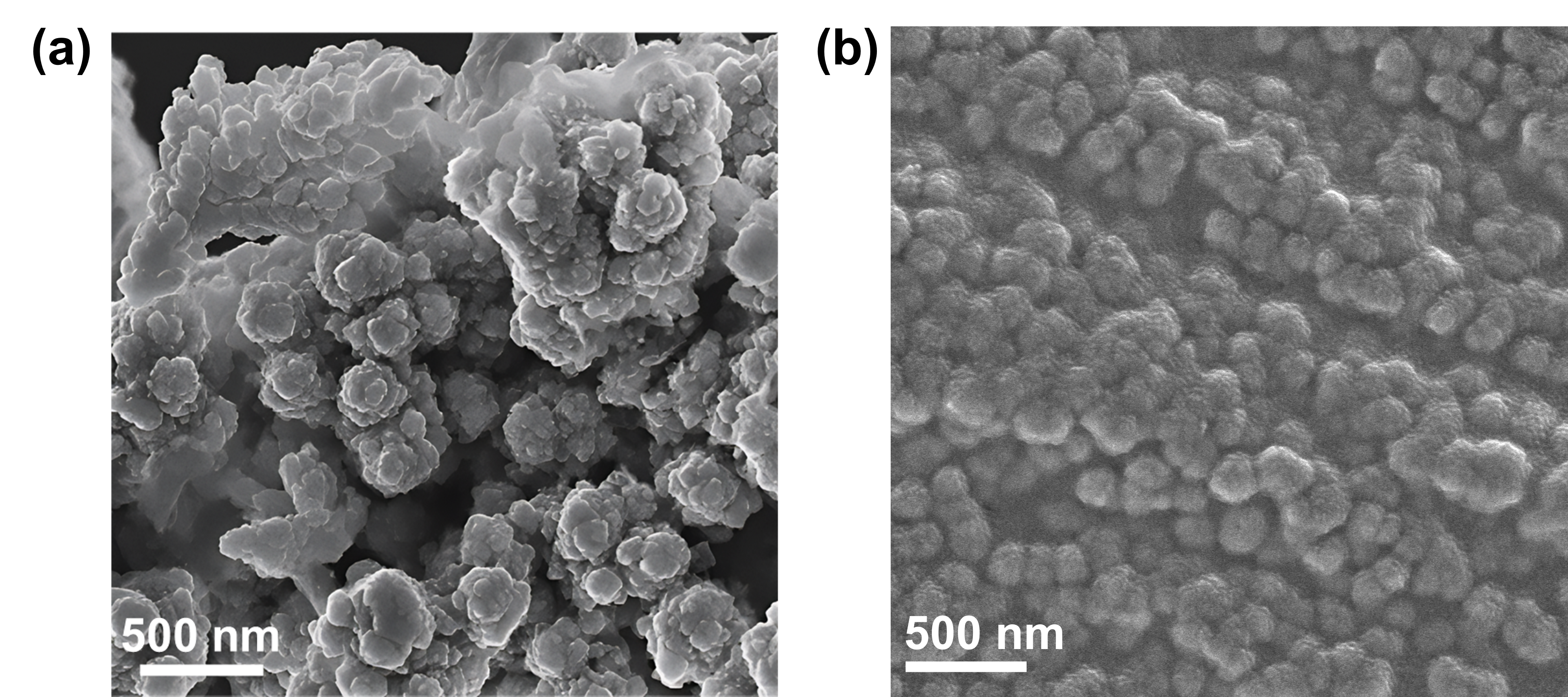


**Figure S26.** SEM images of CuBi-E in CO_2_-saturated 0.1 M KHCO_3_ solution (a) before and (b) after cyclic experiment.

**Figure S27.** XRD patterns of CuBi-E electrodes in CO_2_-saturated 0.1 M KHCO_3_ solution before and after cyclic experiment.


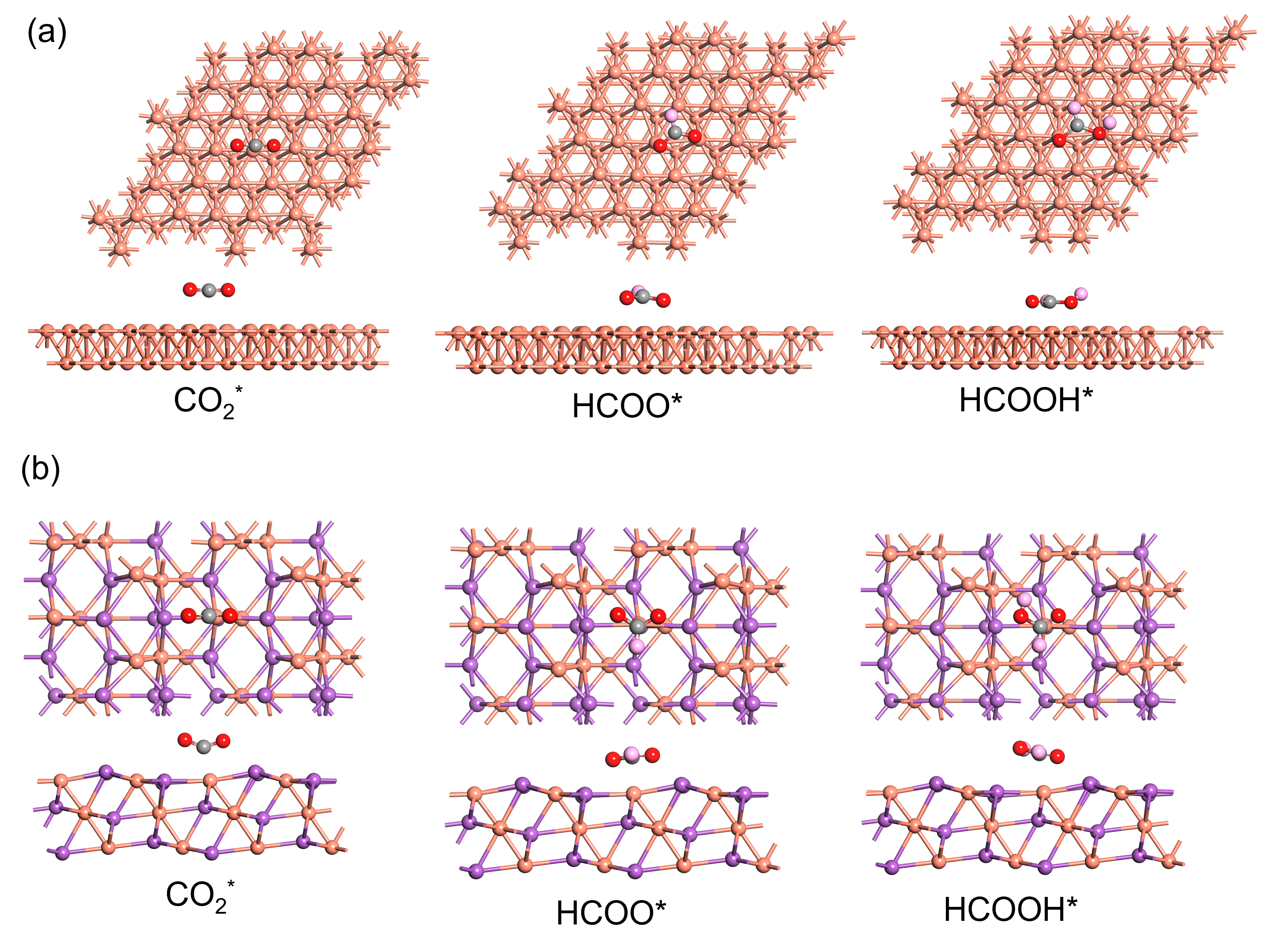


**Figure S28.** Adsorption states of intermediate states on (a) Cu (111), (b) CuBi (012) during the FA generation pathway.


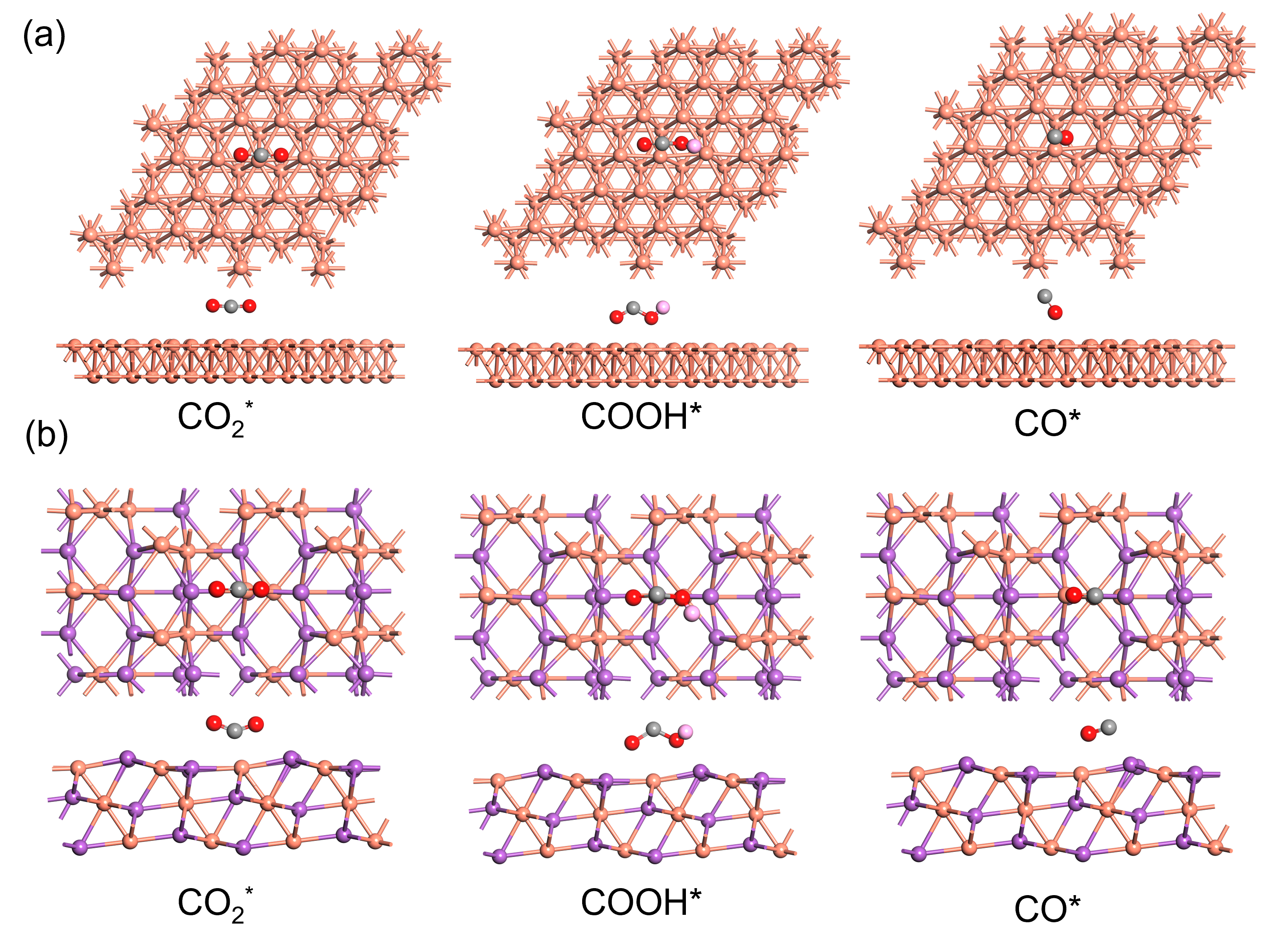


**Figure S29.** Adsorption states of intermediate states on (a) Cu (111), (b) CuBi (012) during the CO generation pathway.


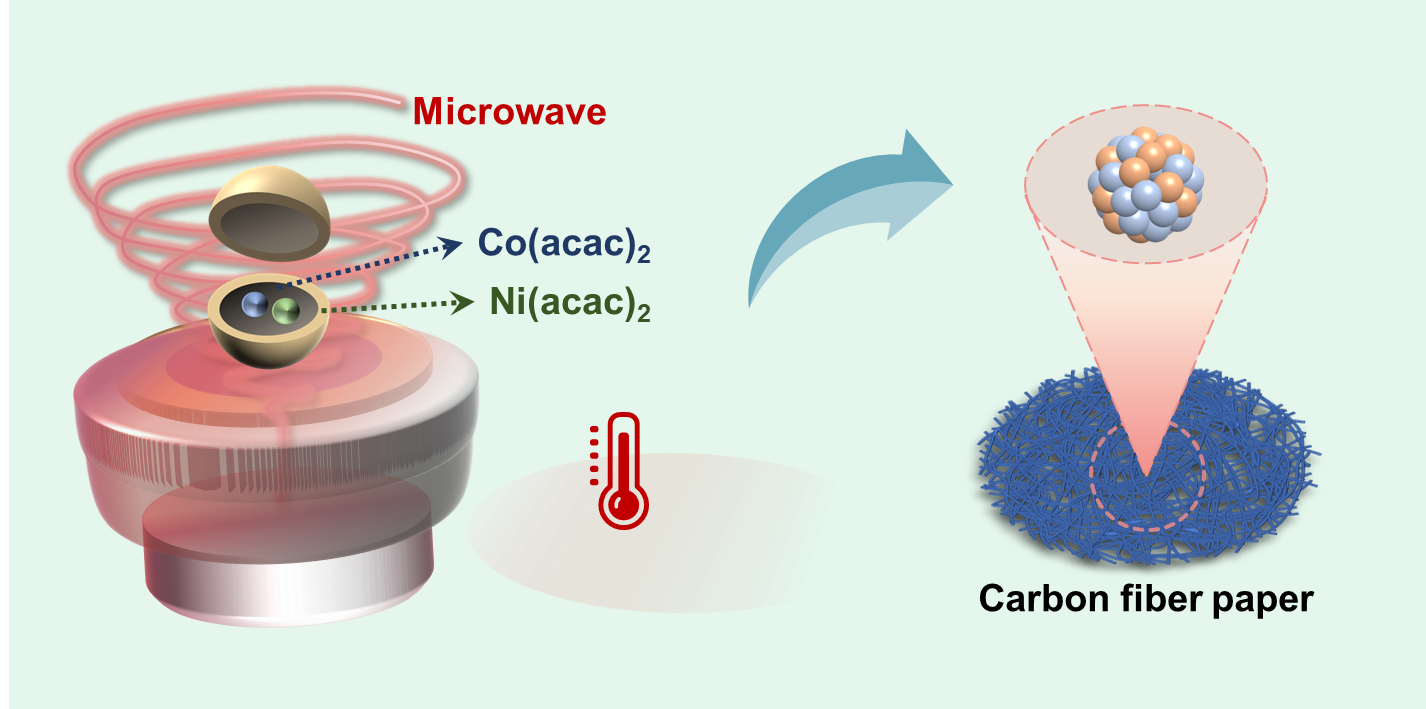


**Figure S30.** Schematic illustration of one-step microwave synthesis of NiCo alloy.

.
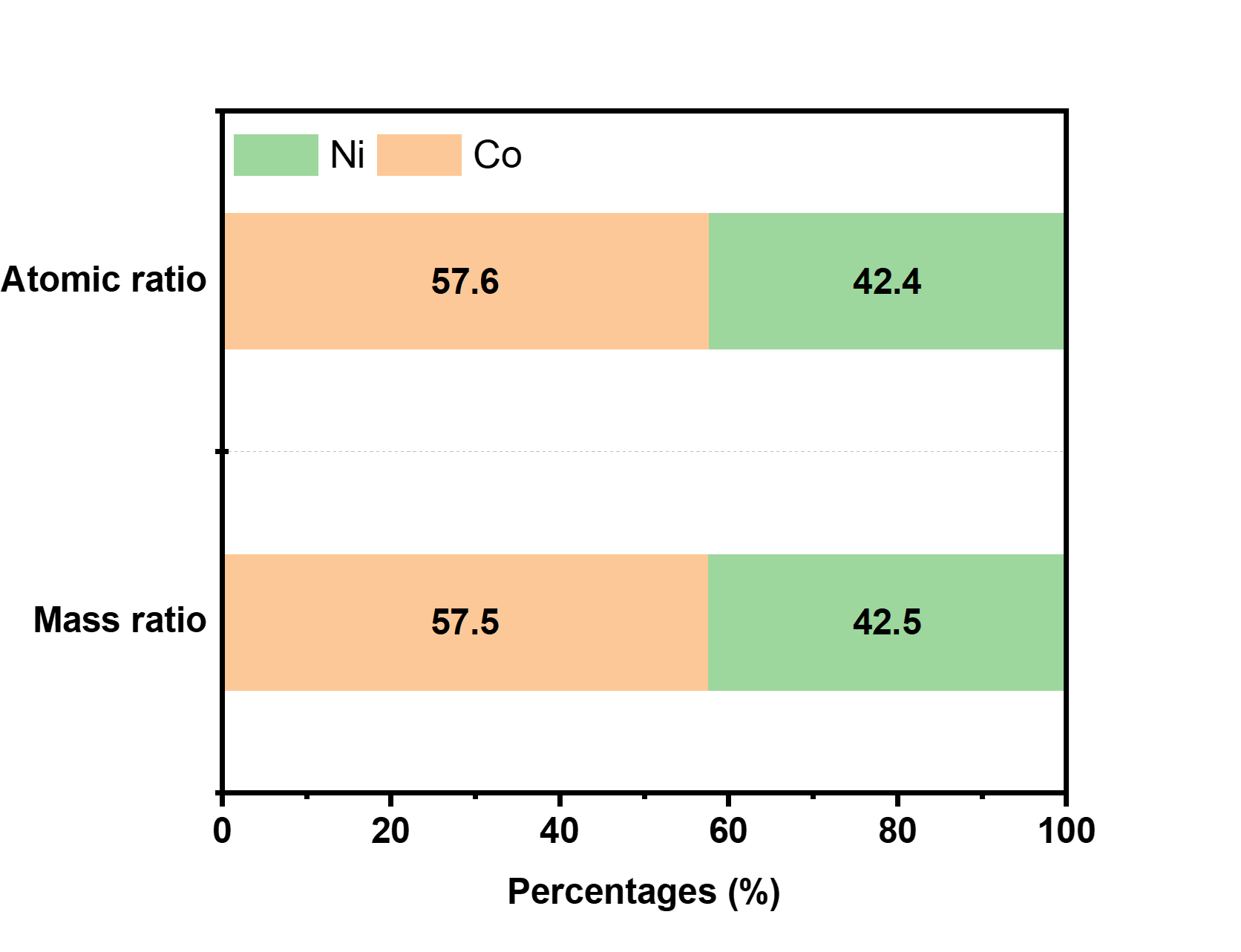


**Figure S31.** The elemental mass fraction in NiCo alloy was measured by ICP-OES.


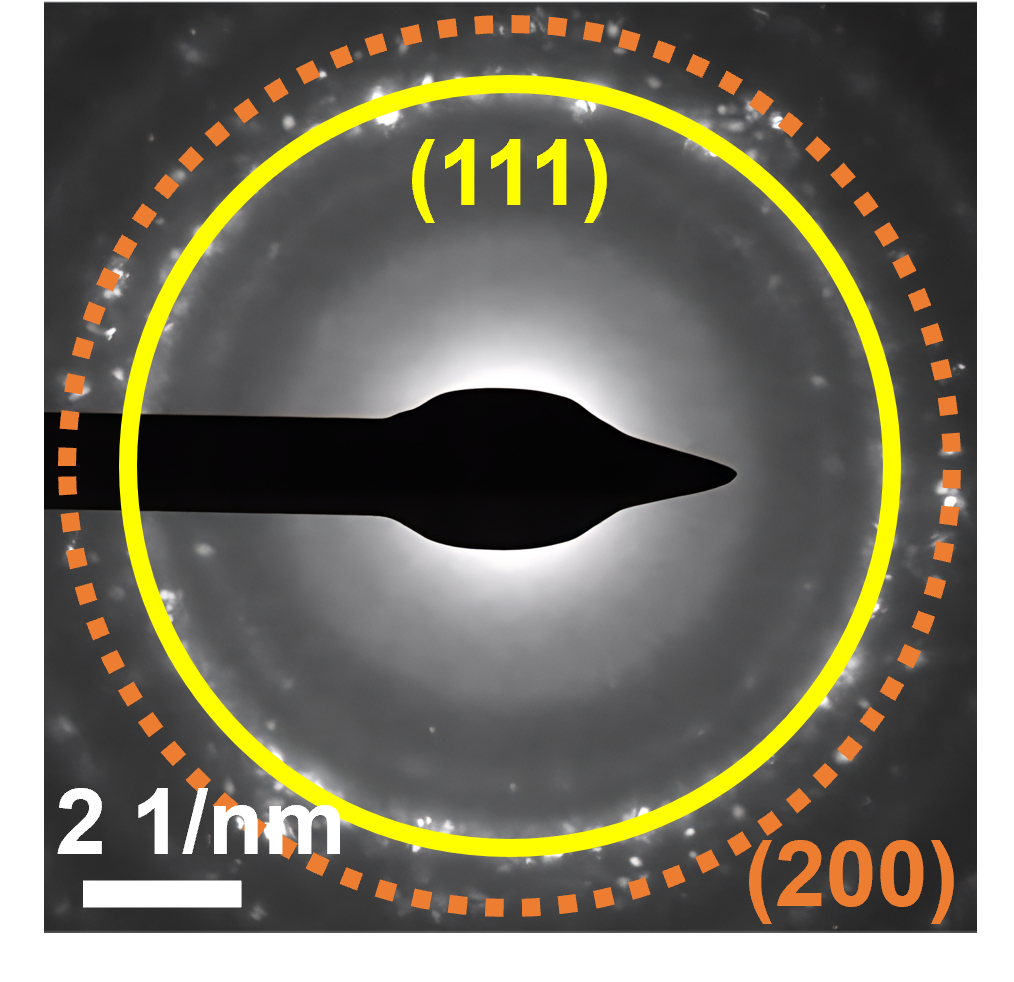


**Figure S32.** SAED image of NiCo alloy.


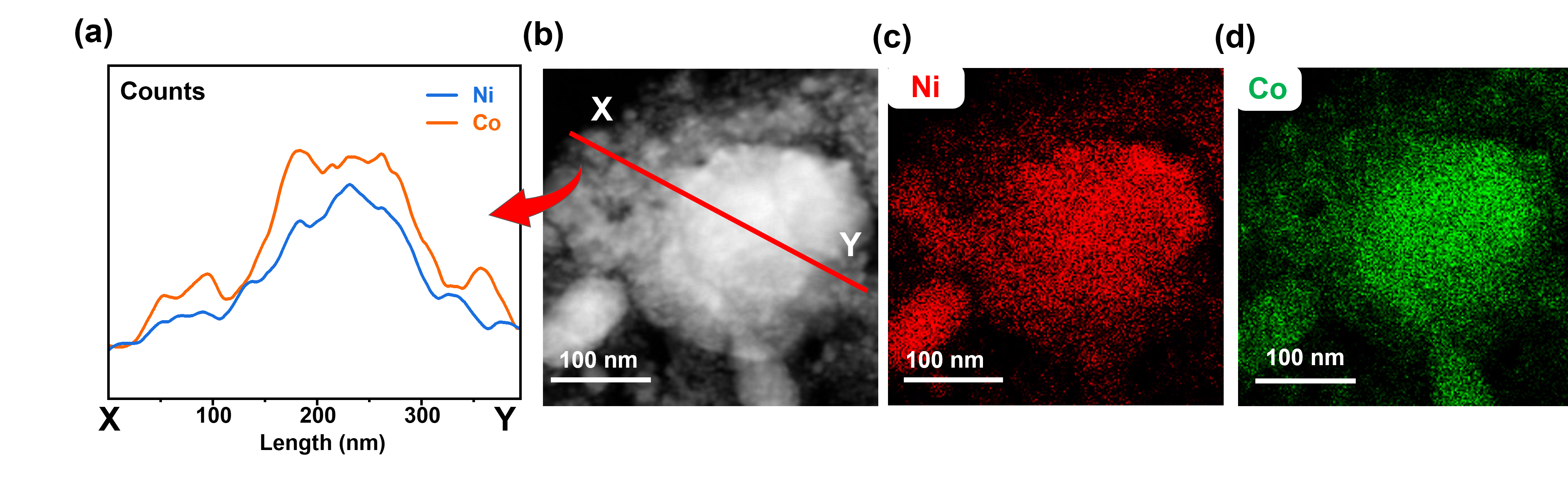


**Figure S33.** (a-d) STEM image and corresponding line scanning profiles and Ni, Co mapping images.


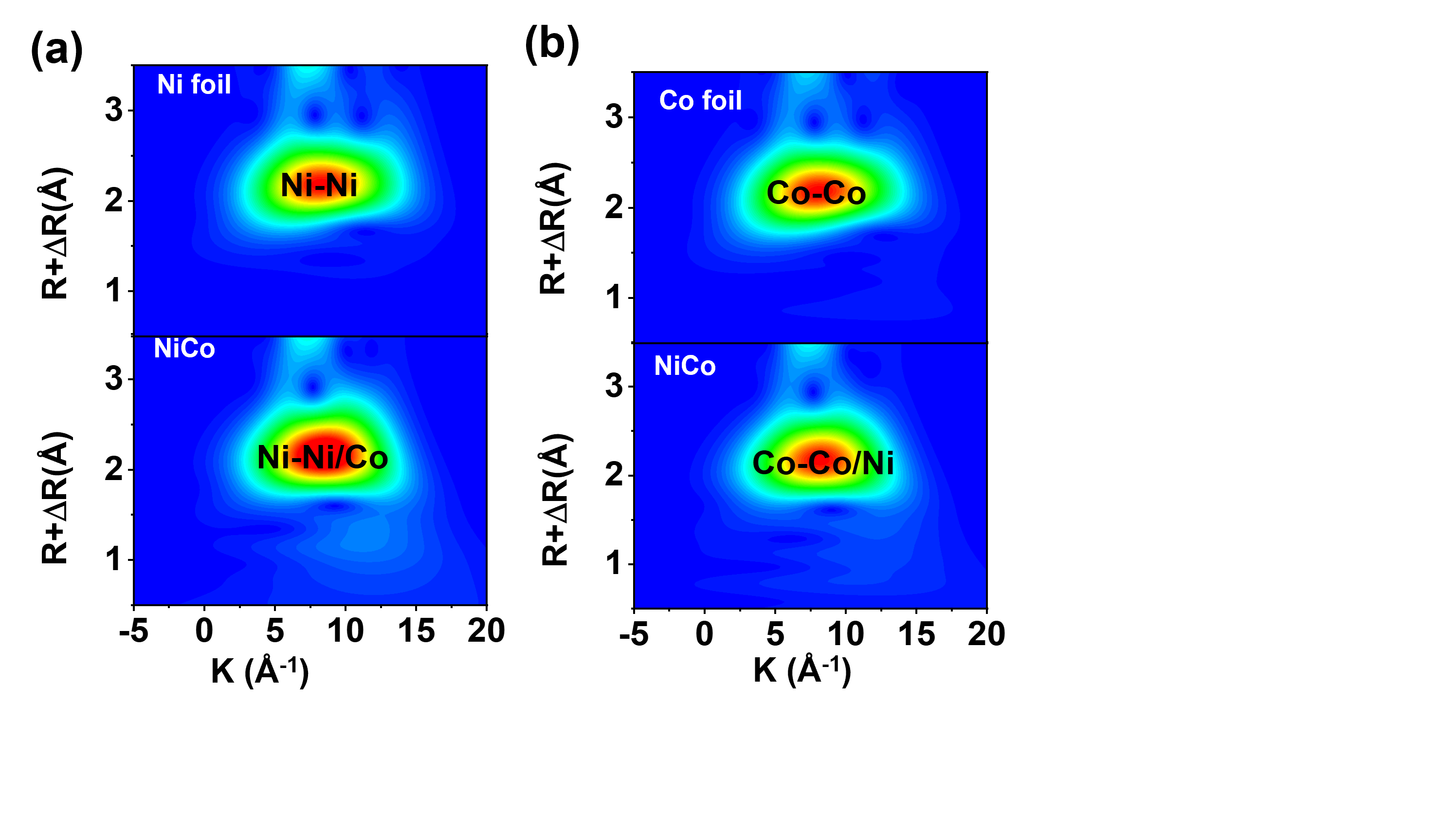


**Figure S34.** (a) WT-EXAFS plots of NiCo alloy and Ni foil. (b) WT-EXAFS plots of NiCo alloy and Co foil.

**Figure S35.** Nyquist plots of NiCo alloy in 1 M KOH solution with and without 1 M methanol.


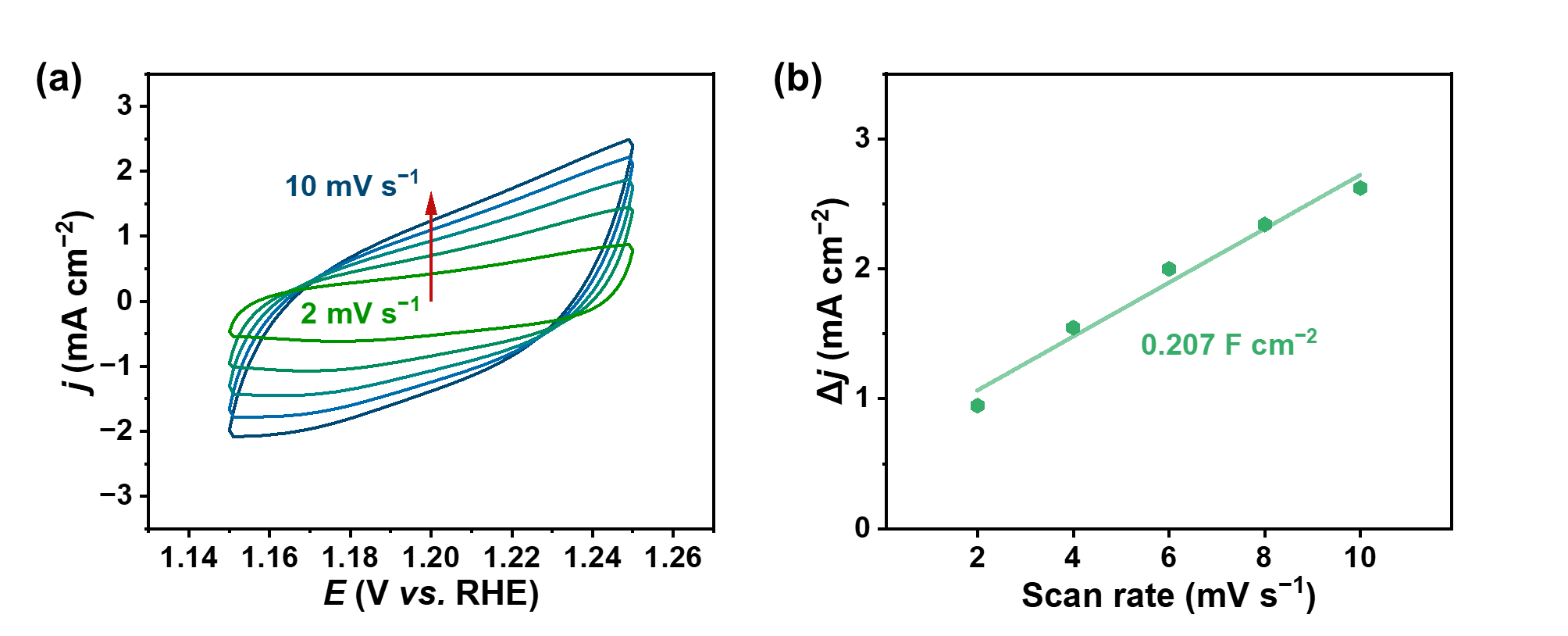


**Figure S36.** (a) CV curves of NiCo alloy at different scan rates. (b) Calculated double-layer capacitance.

**Figure S37.** Formic acid concentration over time during the constant potential test.

**Figure S38.** LSV curves of NiCo alloy in 1 M KOH solution with 1 M methanol before and after long-term constant potential test.


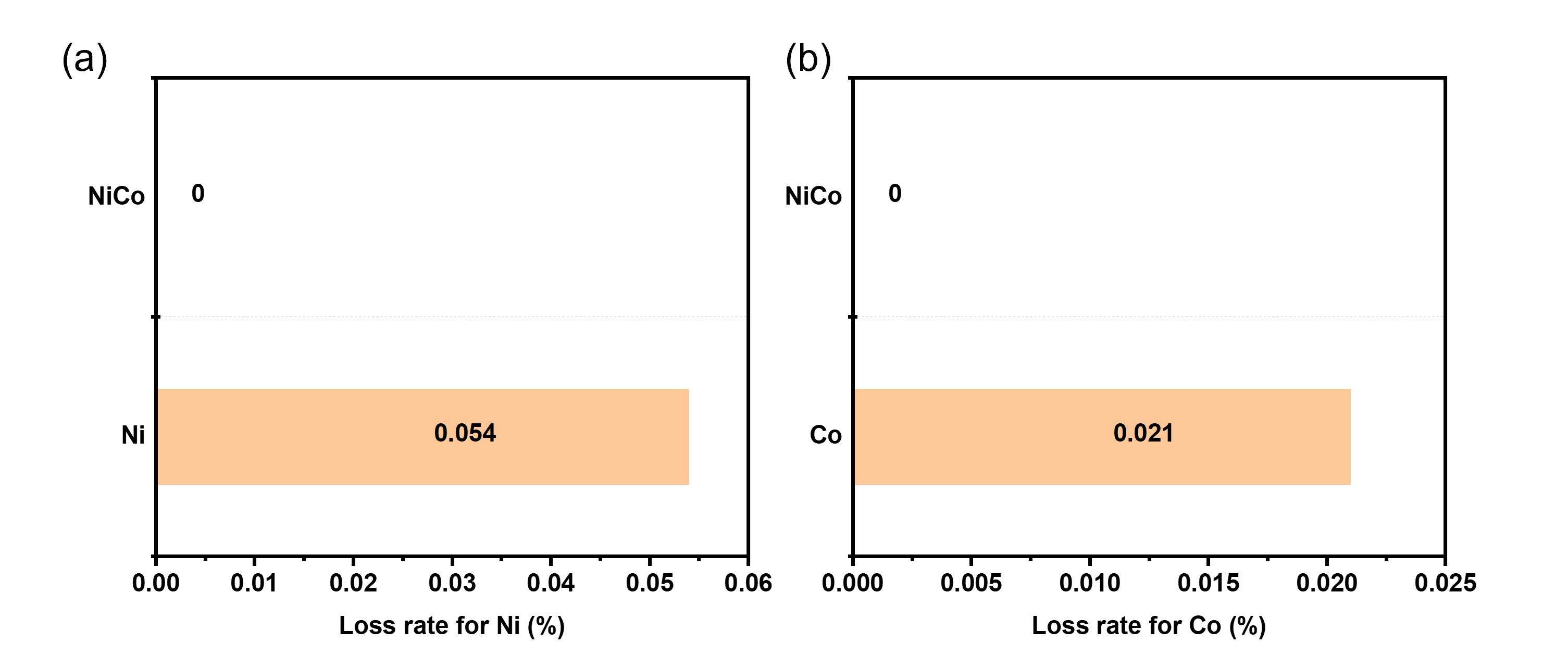


**Figure S39.** The loss rate of Ni and Co from Ni, Co and NiCo catalysts tested by ICP-OES after 20 h MOR.


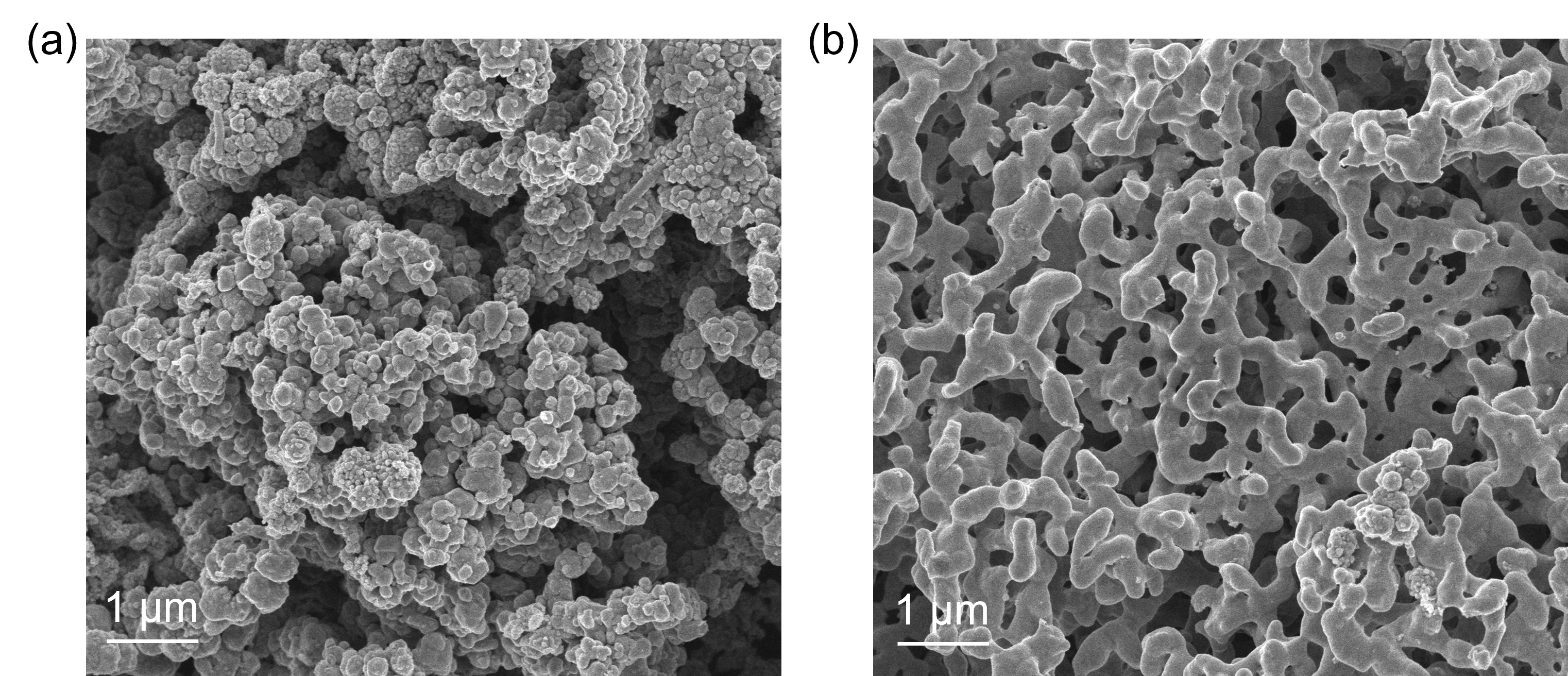


**Figure S40.** SEM images of NiCo catalysts in 1 M KOH solution with 1 M methanol before and after 20 h MOR.

**Figure S41.** XRD patterns of CuBi-E in 1 M KOH solution with 1 M methanol before and after 20 h MOR.


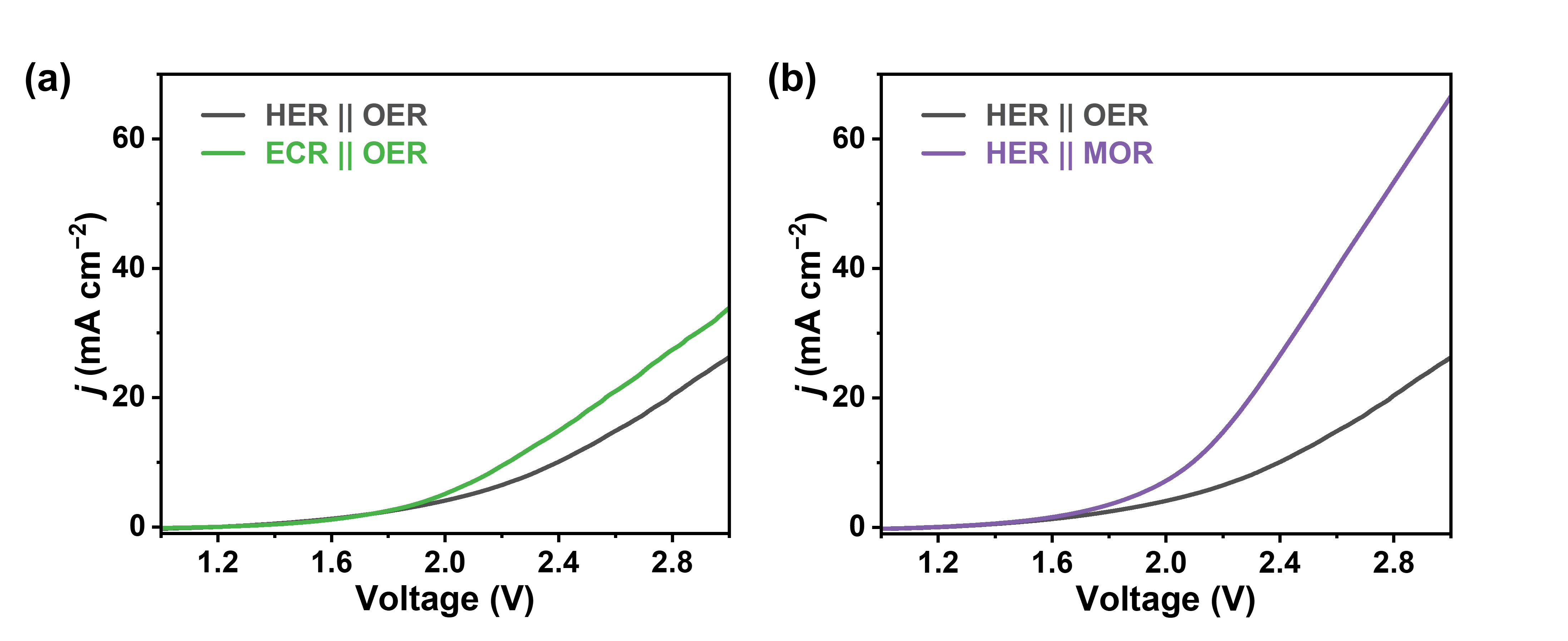


**Figure S42.** LSV curves for (a) ECR||OER and (b) HER||MOR systems compared with that for HER||OER process.

**Figure S43.** CA test curves in the ECR||MOR system at different applied voltages.

**Figure S44.** The formic acid concentration in the cathode and anode during the 20-hour constant potential test in the ECR||MOR system.

**Figure S45.** LSV curves before and after long-term electrocatalytic process in the ECR||MOR system.

**Table S1.** EXAFS fitting parameters at the Co K-edge for NiCo.

|  | Shell | *N^[a]^* | *R*(Å)*^[b]^* | *σ*^2^(Å^2^)*^[c]^* | Δ*E*_0_(eV)*^[d]^* | *R* factor |
| --- | --- | --- | --- | --- | --- | --- |
| Co foil | Co-Co | 12 | 2.49 ± 0.002 | 0.006 ± 0.0003 | 6.7 ± 0.5 | 0.001 |
| NiCo | Co-Co/Ni | 5.9 ± 0.2 | 2.48 ± 0.002 | 0.005 ± 0.0002 | 6.55 ± 0.4 | 0.0017 |

*^a^N*: coordination numbers; *^b^R*: bond distance; *^c^σ*^2^: Debye-Waller factors; *^d^*Δ*E*_0_: the inner potential correction. *R* factor: goodness of fit. *Ѕ*_0_^2^ was set to 0.9 for Co, according to the experimental EXAFS fit of Co foil by fixing CN as the known crystallographic value. For Co foil EXAFS fitting, the data ranges are presented as follows: 3.0 ≤ *k* ≤ 12.5 Å^-1^, 1.2 ≤ *R* ≤ 3.2 Å. The independent point is 11.2, and the number of variables is 4.

**Table S2.** EXAFS fitting parameters at the Cu K edge for BiCu and Ni K-edge for NiCo.

|  | Shell | *N^[a]^* | *R*(Å)*^[b]^* | *σ*^2^(Å^2^)*^[c]^* | Δ*E*_0_(eV)*^[d]^* | *R* factor |
| --- | --- | --- | --- | --- | --- | --- |
| CuBi | Cu-Cu/Bi | 8.4 ± 0.6 | 2.54 ± 0.004 | 0.009 ± 0.0005 | 2.87 ± 0.7 | 0.006 |
| NiCo | Ni-Ni/Co | 4.0 ± 0.3 | 2.49 ± 0.005 | 0.002 ± 0.0006 | 5.72 ± 0.9 | 0.008 |

*^a^N*: coordination numbers; *^b^R*: bond distance; *^c^σ*^2^: Debye-Waller factors; *^d^*Δ*E*_0_: the inner potential correction. *R* factor: goodness of fit. *Ѕ*_0_^2^ was set to 0.85 for Cu, according to the experimental EXAFS fit of Cu foil by fixing CN as the known crystallographic value. For Cu EXAFS fitting, the data ranges are presented as follows: 3.0 ≤ *k* ≤ 12 Å^-1^, 1 ≤ *R* ≤ 3 Å. The independent point is 11.2, and the number of variables is 4. *Ѕ*_0_^2^ was set to 0.85 for Ni, according to the experimental EXAFS fit of Ni foil by fixing CN as the known crystallographic value. For Ni EXAFS fitting, the data ranges are presented as follows: 3.0 ≤ *k* ≤ 12 Å^-1^, 1 ≤ *R* ≤ 3 Å. The independent point is 11.2, and the number of variables is 4.

**References**

[1] Z. Li, Y. Yan, S.-M. Xu, H. Zhou, M. Xu, L. Ma, M. Shao, X. Kong, B. Wang, L. Zheng, H. Duan, Alcohols Electrooxidation Coupled with H_2_ Production at High Current Densities Promoted by a Cooperative Catalyst. *Nat. Commun.* 2022, *13*, 147.

[2] C. Liu, M. Han, C.-L. Chen, J. Yin, L. Zhang, J. Sun, Decorating Phosphorus Anode with SnO_2_ Nanoparticles to Enhance Polyphosphides Chemisorption for High-Performance Lithium-Ion Batteries. *Nano Lett.* **2023**, *23*, 3507-3515.

[3] H. Yousefi-Mashhour, S. Safaeipour, S. Hassani, M. M. Kalantarian, A. Namiranian, Exploring the Potential of Lithium Metal Oxyfluoride, LiMOF, Compounds (M = Mn, Fe, Co, and Ni) for Advanced Li-Ion Battery Applications: A Comprehensive ab Initio Investigation. *J. Phys. Chem. C* **2024**, *128*, 759-767.

[4] M. Ding, Z. Wei, D. Liu, W. Zhao, Q. Lu, Z. Li, Q. Yu, C. Lu, H. Yang, Electronic Modulation Towards MOFs as Template Derived CoP Via Engineered Heteroatom Defect for a Highly Efficient Overall Water Splitting. *J. Energy Chem.* **2025**, *101*, 598-607.

[5] S. Yang, L. Lu, P. Zhan, Z. Si, L. Chen, Y. Zhuang, P. Qin, Amorphous Hetero-Structure Iron/Cobalt Oxyhydroxide with Atomic Dispersed Palladium for Oxygen Evolution Reaction. *Appl. Catal. B* **2024**, *355*, 124213.
